# Biological Function of Prophage-Related Gene Cluster Δ*VpaChn25_RS25055*~Δ*VpaChn25_0714* of *Vibrio parahaemolyticus* CHN25

**DOI:** 10.3390/ijms25031393

**Published:** 2024-01-23

**Authors:** Hui Zhao, Yingwei Xu, Lianzhi Yang, Yaping Wang, Mingyou Li, Lanming Chen

**Affiliations:** 1 Key Laboratory of Quality and Safety Risk Assessment for Aquatic Products on Storage and Preservation (Shanghai), Ministry of Agriculture and Rural Affairs of China, College of Food Science and Technology, Shanghai Ocean University, Shanghai 201306, China; d200300075@st.shou.edu.cn (H.Z.); d220300079@st.shou.edu.cn (Y.X.); d210300069@st.shou.edu.cn (L.Y.); 2 Department of Internal Medicine, Virginia Commonwealth University/McGuire VA Medical Centre, Richmond, VA 23284, USA; yapingwang2016@gmail.com; 3 Key Laboratory of Exploration and Utilization of Aquatic Genetic Resources, Ministry of Education, Shanghai Ocean University, Shanghai 201306, China; myli@shou.edu.cn

**Keywords:** *Vibrio parahaemolyticus*, foodborne pathogen, prophage, transcriptome

## Abstract

*Vibrio parahaemolyticus* is the primary foodborne pathogen known to cause gastrointestinal infections in humans. Nevertheless, the molecular mechanisms of *V. parahaemolyticus* pathogenicity are not fully understood. Prophages carry virulence and antibiotic resistance genes commonly found in *Vibrio* populations, and they facilitate the spread of virulence and the emergence of pathogenic *Vibrio* strains. In this study, we characterized three such genes, *VpaChn25_0713*, *VpaChn25_0714*, and *VpaChn25_RS25055*, within the largest prophage gene cluster in *V. parahaemolyticus* CHN25. The deletion mutants Δ*VpaChn25_RS25055*, Δ*VpaChn25_0713*, Δ*VpaChn25_0714*, and Δ*VpaChn25_RS25055-0713-0714* were derived with homologous recombination, and the complementary mutants Δ*VpaChn25_0713*-com, Δ*VpaChn25_0714*-com, Δ*VpaChn25_RS25055*-com, Δ*VpaChn25_RS25055-0713-0714*-com were also constructed. In the absence of the *VpaChn25_RS25055*, *VpaChn25_0713*, *VpaChn25_0714*, and *VpaChn25_RS25055-0713-0714* genes, the mutants showed significant reductions in low-temperature survivability and biofilm formation (*p* < 0.001). The Δ*VpaChn25_0713*, Δ*VpaChn25_RS25055*, and Δ*VpaChn25_RS25055-0713-0714* mutants were also significantly defective in swimming motility (*p* < 0.001). In the Caco-2 model, the above four mutants attenuated the cytotoxic effects of *V. parahaemolyticus* CHN25 on human intestinal epithelial cells (*p* < 0.01), especially the Δ*VpaChn25_RS25055* and Δ*VpaChn25_RS25055-0713-0714* mutants. Transcriptomic analysis showed that 15, 14, 8, and 11 metabolic pathways were changed in the Δ*VpaChn25_RS25055*, Δ*VpaChn25_0713*, Δ*VpaChn25_0714*, and Δ*VpaChn25_RS25055-0713-0714* mutants, respectively. We labeled the *VpaChn25_RS25055* gene with superfolder green fluorescent protein (sfGFP) and found it localized at both poles of the bacteria cell. In addition, we analyzed the evolutionary origins of the above genes. In summary, the prophage genes *VpaChn25_0713*, *VpaChn25_0714*, and *VpaChn25_RS25055* enhance *V. parahaemolyticus* CHN25’s survival in the environment and host. Our work improves the comprehension of the synergy between prophage-associated genes and the evolutionary process of *V. parahaemolyticus*.

## 1. Introduction

*Vibrio parahaemolyticus* is a Gram-negative, halophilic, and rod-shaped bacterium that is found growing in coastal areas and river–sea junctures at a global scale [1,2]. *V. parahaemolyticus* can cause acute diarrhea, abdominal cramps, vomiting, and fever in humans, and even death [3]. The bacterium was first discovered in Japan in 1950 when the consumption of contaminated semi-dried juvenile sardines caused 272 illnesses and 20 deaths [4]. Since then, outbreaks of the foodborne illness caused by *V. parahaemolyticus* have occurred in many Asian countries, including Bangladesh, China, India, and Malaysia, and then spread to Asia, America, Africa, and Europe [5,6,7]. According to the Mortality Weekly Report of the CDC (Centers for Disease Control and Prevention) of America, *V. parahaemolyticus* causes 45,000 illnesses annually in the United States (https://www.cdc.gov/vibrio/faq.html, accessed on 5 March 2019). Approximately 23.12% of foodborne disease outbreaks in coastal cities in China are associated with *V. parahaemolyticus* [8]. For instance, Luo et al. estimated that the annual incidence rate of *V. parahaemolyticus* gastroenteritis was 183 cases per 100,000 individuals [9]. The hallmark virulence factors associated with *V. parahaemolyticus* include thermostable direct hemolysin (TDH) and TDH-related hemolysin (TRH) [10]. However, some clinical isolates were negative for the two toxins and the type III secretion system (T3SS), indicating that other virulence-associated determinants exist.

Phages, the most abundant biological entities in the biosphere, are viruses that infect bacteria [11,12,13,14,15]. Horizontal gene transfer (HGT) facilitated by prophages strongly influences bacterial evolution by granting them access to novel ecological habitats, including pathogenic traits [16]. For example, there are ~300 genes novel to *V. parahaemolyticus* BB22OP and ~400 genes novel to *V. parahaemolyticus* RIMD2210633. Many of these novel genes are remnants of transposons or phages [17]. Zabala et al. [18] revealed that in the pandemic *V. parahaemolyticus* O3:K6 clonal complex, the presence of a 42 kb prophage led to a variant, and *V. parahaemolyticus* O3:K6 carrying this prophage displayed an ultraviolet radiation sensitivity that was 7–15 times higher. The prophages in *V. parahaemolyticus*, such as *VP06*, *Vp882*, and *Vp58.5*, contribute to various functions, including increased sensitivity to ultraviolet radiation, DNA methylase activity, quorum sensing, and improved resistance to environmental stress [19]. Yang and co-workers [20] found that *V. parahaemolyticus* carrying prophages 12B12, VEJphi, VCY_phi, and VFJ caused acute hepatopancreatic necrosis disease (AHPND) in shrimps. Prophages are essential for the biological properties of bacterial hosts; thus, it is necessary to recognize them accurately and understand their function via nucleotide sequence analysis [21].

Our previous studies isolated a *V. parahaemolyticus* CHN25 strain (serotype: O5: KUT) of aquatic animal origin, followed by identification and characterization [22,23,24,25,26]. It was found that prophage gene clusters were present in chromosome 1 (3,416,467 bp) of the *V. parahaemolyticus* CHN25 genome [24], within which the biological functions of two genes *VpaChn25_0734* (543 bp) and *VpaChn25_0724* (294 bp) have been characterized recently. The *VpaChn25_0734* gene encodes a predicted phage virion morphogenetic protein with conserved structural domains belonging to the Phage_tail_S superfamily, and the *VpaChn25_0724* gene encodes an unknown hypothetical protein without conserved structural domains [15,25]. Sequence analysis revealed that the *VpaChn25_RS25055*, *VpaChn25_0713*, and *VpaChn25_0714* genes belong to the same prophage gene cluster, but their biological functions are unknown. Therefore, we focused on these unknown genes in this study. To facilitate an improved understanding of the biological functions of unknown protein-encoding genes in the prophage clusters retained in the *V. parahaemolyticus* genome, herein, we investigated the impact of the *VpnChn25_RS25055*, *VpnChn25_0713*, and *VpnChn25_0714* genes on the survival of the host for the first time. The objectives of this study were (1) to construct three single-gene mutants, Δ*VpaChn25_RS25055*, Δ*VpaChn25_0713*, and Δ*VpaChn25_0714*, as well as a triple-gene mutant, Δ*VpaChn25_RS25055-0713-0714*, using the homologous recombination technique. Meanwhile, the complementary mutants Δ*VpaChn25_RS25055*-com, Δ*VpaChn25_0713*-com, Δ*VpaChn25_0714*-com, and Δ*VpaChn25_RS25055-0713-0714*-com were also established; (2) to evaluate the motility, growth, cell toxicity, and biofilm formation of the Δ*VpaChn25_RS25055*, Δ*VpaChn25_0713*, Δ*VpaChn25_0714*, and Δ*VpaChn25_RS25055-0713-0714* mutants compared to *V. parahaemolyticus* CHN25 wild type (WT) and the complementary mutants; (3) to elucidate the molecular mechanisms underlying the changed phenotypes of Δ*VpaChn25_0713*, Δ*VpaChn25_0714*, Δ*VpaChn25_RS25055*, and Δ*VpaChn25_RS25055-0713-0714* mutants with comparative transcriptomic analysis; and (4) to label the prophage gene *VpaChn25_RS25055* with sfGFP and monitor its position in the cell. Our findings could enhance the comprehension of *V. parahaemolyticus* genome evolution and pathogenicity.

## 2. Results

### 2.1. Prophage-Related Genes VpaChn25_RS25055, VpaChn25_0713, and VpaChn25_0714 in V. parahaemolyticus CHN25

**Figure 1:**
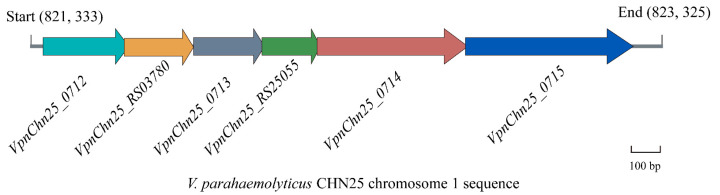
Organization of the *V. parahaemolyticus CHN25* prophage genes *VpnChn25_0713*, *VpnChn25_0714*, and *VpnChn25_RS25055*.

The *V. parahaemolyticus* CHN25 genome contains a large prophage-like gene cluster, which shows high sequence similarity with the *Vibrio* phage martha 12B12 (33, 277 bp, GenBank accession no. HQ_316581) containing 50 predicted genes [24]. Of the twenty-four genes present in chromosome 1 (3,416,467 bp) of the *V. parahaemolyticus* CHN25 genome, seven coded for phage proteins, eight encoded predictive regulators, and nine coded for hypothetical proteins with unknown functions in the current databases [25]. Of these unknown genes, sequence analysis showed that the *VpaChn25_0713* gene encodes a hypothetical protein that contains a conserved structural domain of the ku superfamily. The *VpaChn25_0714*- and *VpaChn25_RS25055*-encoding proteins had no hits against any conserved structural domains. Meanwhile, *VpaChn25_0713* has a 29 bp overlap with *VpaChn25_RS25055*, and *VpaChn25_RS25055* has a 4 bp overlap with *VpaChn25_0714* (Figure 1).

### 2.2. Deletion and Reverse Complementation of the VpaChn25_RS25055, VpaChn25_0713, and VpaChn25_0714 Genes in V. parahaemolyticus CHN25 Genome

Unmarked in-frame single-gene-deletion mutants Δ*VpaChn25_RS25055*, Δ*VpaChn25_0713*, and Δ*VpaChn25_0714*, as well as the triple-deletion mutant Δ*VpaChn25_RS25055-0713-0714*, were derived with homologous recombination methods. For instance, for the construction of the Δ*VpaChn25_0713* mutant, two primer pairs, *VpaChn25_0713*-up-F/R and *VpaChn25_0713*-down-F/R (Table 1), were designed to target upstream (454 bp) and downstream (322 bp) sequences of the *VpaChn25_0713* gene in the *V. parahaemolyticus* CHN25 genome, respectively. The upstream and downstream sequences were retrieved by the polymerase chain reaction (PCR) and subsequently cloned into pDS132. Subsequently, the ligated DNA was transfected into *E. coli* DH5*α* λpir-competent cells, and subsequent screening identified the positive transformants as obtaining the recombinant vector pDS132+*VpaChn25_0713*. The recombinant vector was introduced into *E. coli β*2155-competent cells, followed by conjugation with *V. parahaemolyticus* CHN25. Positive exconjugants were obtained using the two-step allele exchange approach [25]. The 234 bp *VpaChn25_0713* deletion was verified by the DNA sequencing and PCR analyses (Figure S1). Likewise, we used the same method to construct the deletion mutants Δ*VpaChn25_0714*, Δ*VpaChn25_RS25055*, and Δ*VpaChn25_RS25055-0713-0714* (Figures S2–S4). Sequencing chromatographs of *V. parahaemolyticus* CHN25 WT and Δ*VpaChn25_0713*, Δ*VpaChn25_RS25055*, Δ*VpaChn25_0714*, and Δ*VpaChn25_RS25055-0713-0714* mutants are presented in Figure S5.

Subsequently, four reverse mutants, Δ*VpaChn25_RS25055*-com, Δ*VpaChn25_0713*-com, Δ*VpaChn25_0714*-com, and Δ*VpaChn25_RS25055-0713-0714*-com, were also successfully constructed, respectively. For example, for the construction of the complementary mutant Δ*VpaChn25_0713*-com, the 234 bp *VpaChn25_0713* was subjected to amplification via the PCR assay, followed by cloning into pMMB207 (9076 bp). The ligated DNA was introduced into *E. coli* DH5*α*, and positive transformants were screened to obtain pMMB207+*VpaChn25_0713*. Electrotransformation introduced the recombinant vector into the Δ*VpaChn25_0713* mutant, generating the reverse mutant Δ*VpaChn25_0713*-com. Confirmation of this mutant was carried out using the methods previously mentioned (Figure S6). Similarly, the same method was used to construct Δ*VpaChn25_0714*-com, Δ*VpaChn25_RS25055*-com, and Δ*VpaChn25_RS25055-0713-0714*-com (Figures S7–S9).

### 2.3. Survival of ΔVpaChn25_0713, ΔVpaChn25_0714, ΔVpaChn25_RS25055, and ΔVpaChn25_RS25055-0713-0714 Mutants at Different Temperatures and pH Conditions

**Figure 2:**
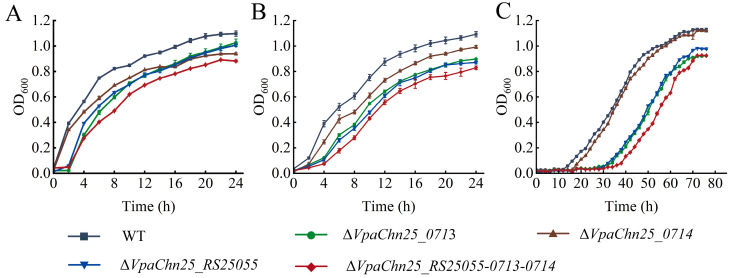
Survival of the *V. parahaemolyticus* CHN25 (WT), Δ*VpaChn25_RS25055*, Δ*VpaChn25_0713*, Δ*VpaChn25_0714*, and Δ*VpaChn25_RS25055-0713-0714* strains in the TSB medium (pH 8.5, 3% NaCl) at different temperatures. (**A**–**C**) 37, 25, and 15 °C, respectively.

**Figure 3:**
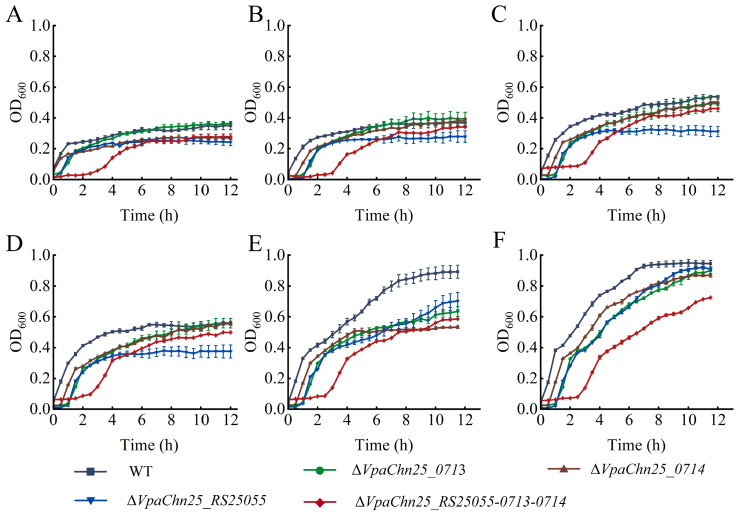
Survival of the *V. parahaemolyticus* CHN25 WT, Δ*VpaChn25_RS25055*, Δ*VpaChn25_0713*, Δ*VpaChn25_0714*, and Δ*VpaChn25_RS25055-0713-0714* strains in the TSB medium (pH 8.5, 3% NaCl) at different pH conditions. (**A**–**F**) pH 5.5, pH 6.0, pH 6.5, pH 7.0, pH 7.5, and pH 8.0, respectively.

*V. parahaemolyticus* can grow in a broad range of environmental conditions such as pH 5.0–9.0 and NaCl 0.5–3% [27]. To investigate the influence of the deletion of the *VpaChn25_RS25055*, *VpaChn25_0713*, *VpaChn25_0714*, and *VpaChn25_RS25055-0713-0714* genes on *V. parahaemolyticus* CHN25 survival, growth curves of the WT strain, four deletion mutants, and four complementary mutants were determined at varying temperatures (15, 25, and 37 °C) and pH conditions (pH 5.5 to 8.0), and the data are presented in Figure 2, Figure 3, Figure S10, and Figure S11, respectively.

As shown in Figure 2A, at 37 °C, the Δ*VpaChn25_0713*, Δ*VpaChn25_RS25055*, and Δ*VpaChn25_RS25055-0713-0714* mutants grew in TSB medium (pH 8.5, 3% NaCl) with a delay phase (DP) of 2 h when compared with the WT strain. The maximum OD_600_ value of Δ*VpaChn25_RS25055-0713-0714* (0.89 ± 0.01) was significantly lower than that of the WT strain (1.10 ± 0.02) (*p* < 0.01).

At 25 °C, the maximum OD_600_ value of Δ*VpaChn25_0713*, Δ*VpaChn25_RS25055*, and Δ*VpaChn25_RS25055-0713-0714* mutants was significantly lower than that of the WT strain (1.09 ± 0.02) (*p* < 0.01) (Figure 2B).

At 15 °C, the growth of the WT and four mutant strains were all delayed (Figure 2C). The WT strain entered the logarithmic phase (LP) after 8 h and the stationary phase (SP) after 60 h with a maximum OD_600_ value of 1.13 ± 0.01. The Δ*VpaChn25_0713* mutant grew more slowly during the first 30 h of incubation and entered the LP after 32 h and the SP after 68 h with the maximum OD_600_ value of 0.91 ± 0.01. Similarly, the lag phases of the Δ*VpaChn25_RS25055* and Δ*VpaChn25_RS25055-0713-0714* mutants were 3.75-fold and 4.75-fold longer than that of the WT strain, respectively (Figure 2C).

These results indicated that the *VpaChn25_RS25055* and *VpaChn25_0713* genes could enhance the adaptability of *V. parahaemolyticus* CHN25 for colder conditions. Growth of Δ*VpaChn25_RS25055-0713-0714* was more strongly inhibited than the single-gene-deletion mutants Δ*VpaChn25_RS25055*, Δ*VpaChn25_0713*, and Δ*VpaChn25_0714* at 37 °C, 25 °C, and 15 °C, indicating a positively superposed regulation of the *VpaChn25_RS25055*, *VpaChn25_0713*, and *VpaChn25_0714* genes on the growth of *V. parahaemolyticus* CHN25.

*V. parahaemolyticus* survives the stomach’s harsh acidic environment and establishes intestinal colonization in the host [28]. When consumed with raw, undercooked, or mishandled seafood, *V. parahaemolyticus* is challenged by the very low pH environment of the human stomach (which is normally between 1–3 but can rise above 6.0 after consumption of the food) and reaches the human gastrointestinal tract, where it can cause gastroenteritis [22]. Therefore, we studied the growth of the WT and the four mutants in TSB (3% NaCl) at pH values between 5.5 and 8.0, and the results are shown in Figure 3.

Under acidic (pH 5.5–6.5) and neutral (pH 7.0) conditions, the growth of the WT strain and four mutant strains were greatly inhibited, with the maximum OD_600_ values below 0.6 at the SP (Figure 3A–D). Notably, the maximum OD_600_ value of the Δ*VpaChn25_RS25055* mutant was significantly lower than the WT strain in acidic and neutral conditions (*p* < 0.01). Interestingly, the Δ*VpaChn25_RS25055-0713-0714* mutant grew in the TSB medium with DPs of 2.5 h, 3.0 h, 3.0 h, and 2.5 h when compared with the WT strain at pH 5.5, pH 6, pH 6.5, and pH 7.0, respectively (*p* < 0.01).

Under alkaline conditions, the maximum biomass of the Δ*VpaChn25_RS25055*, Δ*VpaChn25_0713*, Δ*VpaChn25_0714*, and Δ*VpaChn25_RS25055-0713-0714* mutants was significantly lower than WT at pH 7.5 (*p* < 0.01) (Figure 3E). At pH 8.0, the growth of the Δ*VpaChn25_RS25055-0713-0714* mutant was still poor, showing a maximum biomass OD_600_ value of 0.75 ± 0.01 (Figure 3F).

It was observed that both *VpaChn25_0713* and *VpaChn25_0714* genes are beneficial to *V. parahaemolyticus* CHN25 survival at pH 7.0–8.0, while the *VpaChn25_RS25055* gene can amplify *V. parahaemolyticus* CHN25 persistence at pH 5.5–8.0. Interestingly, the LP of Δ*VpaChn25_RS25055-0713-0714* was significantly extended compared to the Δ*VpaChn25_RS25055*, Δ*VpaChn25_0713*, and Δ*VpaChn25_0714* mutants at pH 5.5–8.0, indicating a positively superposed regulation of *VpaChn25_RS25055*, *VpaChn25_0713*, and *VpaChn25_0714* genes on the acid tolerance of *V. parahaemolyticus* CHN25.

### 2.4. Swimming Motility of the ΔVpaChn25_RS25055, ΔVpaChn25_0713, ΔVpaChn25_0714, and ΔVpaChn25_RS25055-0713-0714 Mutants

**Figure 4:**
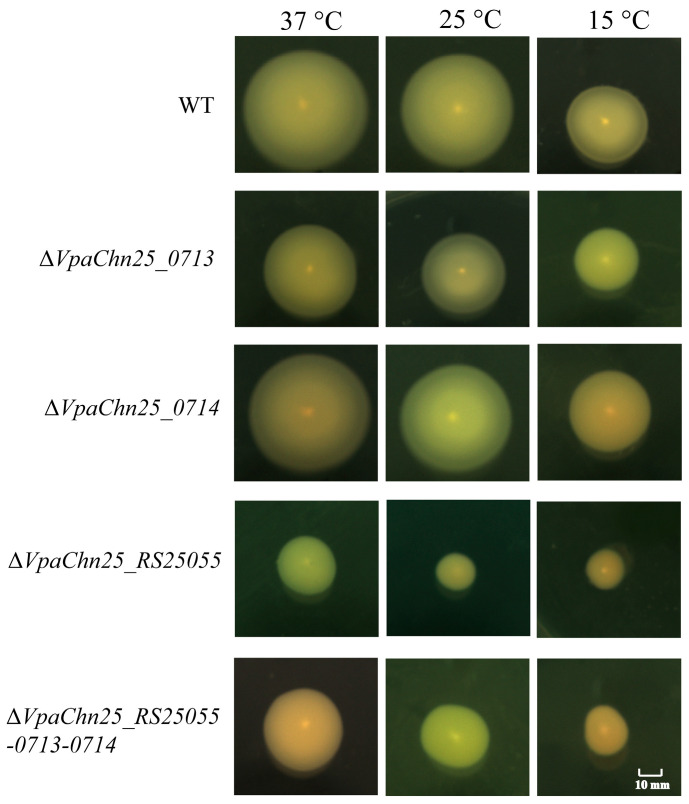
Swimming motility of the *V. parahaemolyticus* CHN25 WT, Δ*VpaChn25_RS25055*, Δ*VpaChn25_0713*, Δ*VpaChn25_0714*, and Δ*VpaChn25_RS25055-0713-0714* strains at different temperatures. The strains were individually incubated in a semi-solid TSB medium containing 0.25% agar at 37 °C, 25 °C, and 15 °C, respectively.

Motility has been identified as an essential virulence factor for the survival and colonization of *V. parahaemolyticus* [29]. Herein, swimming of the WT strain, four mutants, and four complementary mutants were examined at different temperatures; the results are presented in Figure 4 and Figure S12.

When the strains were separately incubated in semi-solid TSB containing 0.25% agar at 37 °C, the Δ*VpaChn25_0713* mutant swam (1.68 ± 0.05 cm) remarkably slower than the WT strain (3.37 ± 0.14 cm) (*p* < 0.01) (Figure 4). A similar case was observed under lower temperatures (Figure 4). This highlighted that *VpaChn25_0713* gene deletion markedly suppressed the motility of *V. parahaemolyticus* CHN25. 

Similarly, the Δ*VpaChn25_RS25055* mutant also swam significantly slower than the WT strain at 15, 25, and 37 °C, respectively (*p* < 0.01) (Figure 4).

The swimming diameters of the Δ*VpaChn25_RS25055-0713-0714* mutant were 1.60 ± 0.07 cm, 1.46 ± 0.18 cm, and 1.12 ± 0.09 cm at 37, 25, and 15 °C, respectively, which were 0.47-fold, 0.56-fold, and 0.42-fold smaller than those of the WT strain (*p* < 0.001) (Figure 4).

In contrast, as depicted in Figure 4, no obvious differences in swimming circles were found between the Δ*VpaChn25_0714* mutant and the WT strain at 15, 25, and 37 °C (*p* > 0.05).

Taken together, these findings demonstrated that a deficiency in motility was induced by *VpaChn25_RS25055*, *VpaChn25_0713*, *and VpaChn25_RS25055-0713-0714* deletion in *V. parahaemolyticus* CHN25. Interestingly, when the strains were separately incubated in semi-solid TSB containing 0.25% agar, the swimming diameters of Δ*VpaChn25_RS25055* were significantly lower than those of Δ*VpaChn25_0713* and Δ*VpaChn25_0714* at 15, 25, and 37 °C (*p* < 0.05).

### 2.5. Biofilm Formation of the ΔVpaChn25_RS25055, ΔVpaChn25_0713, ΔVpaChn25_0714, and ΔVpaChn25_RS25055-0713-0714 Mutants

**Figure 5:**
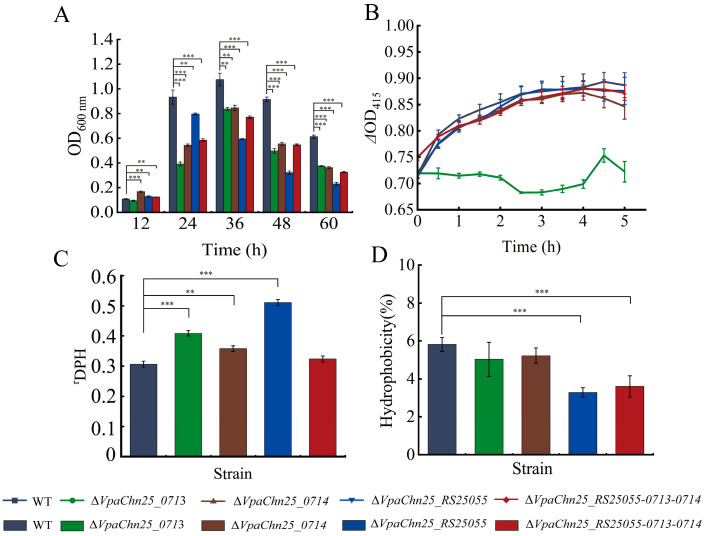
Biofilm formation (**A**), internal membrane permeability (**B**), fluidity (**C**), and hydrophobicity (**D**) of the *V. parahaemolyticus* CHN25 (WT), Δ*VpaChn25_RS25055*, Δ*VpaChn25_0713*, Δ*VpaChn25_0714*, and Δ*VpaChn25_RS25055-0713-0714* strains. DPH: 1, 6-diphenyl-1,3,5-hexatriene. ** *p* < 0.01, *** *p* < 0.001.

*V. parahaemolyticus* can produce adherence factors that facilitate surface attachment and promote biofilm formation, thereby increasing its environmental survival, infectivity, and transmission [30]. Herein, biofilm formation of the WT, four deletion mutants, and four complementary mutants were analyzed by crystalline violet staining at 37 °C for 60 h. The data are presented in Figure 5A and Figure S13A. All strains showed similar biofilm development, maturation, and diffusion stages, but the maximum biofilm biomass formed by the four mutants was remarkably smaller than the WT strain (*p* < 0.001).

As shown in Figure 5A, at 0 to 12 h, the biofilm of the WT strain formed slowly; at 12 to 36 h, it increased rapidly and reached the maximum biomass (OD _600_ = 1.074 ± 0.05) at 36 h; at 36 to 60 h, the biofilm decreased sharply (OD _600_ = 0.614 ± 0.01), which may have resulted from nutrient depletion and accumulation of metabolic waste in the orifice plates.

Compared to the WT strain, the Δ*VpaChn25_0713* mutant showed significantly slower biofilm formation at all stages (*p* < 0.01), reaching maximum biofilm formation at 36 h, which was 0.78-fold less than that of the WT strain (Figure 5A). Similar cases were observed for Δ*VpaChn25_0714* and Δ*VpaChn25_RS25055-0713-0714* (Figure 5A).

In addition, in the absence of the *VpaChn25_RS25055* gene, the biofilm formed by *V. parahaemolyticus* reached the maximum biomass at 24 h, which was 0.74-fold less than that of WT (Figure 5A).

These findings demonstrated that the absence of the *VpaChn25_RS25055*, *VpaChn25_0713*, *VpaChn25_0714*, and *VpaChn25_RS25055-0713-0714* genes led to a decrease in biofilm formation of *V. parahaemolyticus* CHN25. Notably, the maximum biofilm formation of the Δ*VpaChn25_RS25055-0713-0714* mutant was significantly less than those of the Δ*VpaChn25_0713* and Δ*VpaChn25_0714* mutants (*p* < 0.01), indicating the positively superposed regulation of *VpaChn25_RS25055*, *VpaChn25_0713*, and *VpaChn25_0714* genes on the biofilm formation of *V. parahaemolyticus* CHN25.

### 2.6. Cell Surface Hydrophobicity, Cell Membrane Permeability, and Fluidity of the ΔVpaChn25_RS25055, ΔVpaChn25_0713, ΔVpaChn25_0714, and ΔVpaChn25_RS25055-0713-0714 Mutants

Cell membranes serve as selective semi-permeable barriers whose integrity, fluidity, and selective permeation control the movement of various substances, playing a pivotal role in microbial growth and pathogenicity [31]. Based on the above results, we further asked whether the deletion of the *VpaChn25_RS25055*, *VpaChn25_0713*, and *VpaChn25_0714* genes would influence bacterial cell membrane structure (Figure 5 and Figure S13). 

o-nitrophenyl-*β*-D galactopyranoside (ONPG) was used as a probe to examine the cell inner membrane permeability of the strains. As shown in Figure 5B, no apparent differences in cell inner membrane permeability were found between the WT strain and the Δ*VpaChn25_RS25055*, Δ*VpaChn25_0714*, and Δ*VpaChn25_RS25055-0713-0714* mutants, but the inner membrane permeability of Δ*VpaChn25_0713* was significantly reduced (*p* < 0.001).

As displayed in Figure 5C, the cell membrane fluidity of the Δ*VpaChn25_RS25055*, Δ*VpaChn25_0713*, and Δ*VpaChn25_0714* mutants were 1.65-fold, 1.32-fold, and 1.13-fold higher than those of the WT strain, respectively. Additionally, the cell surface hydrophobicity of Δ*VpaChn25_RS25055* and Δ*VpaChn25_RS25055-0713-0714* was 0.56-fold and 0.62-fold lower than that of WT, respectively (Figure 5D).

### 2.7. Interaction between the ΔVpaChn25_RS25055, ΔVpaChn25_0713, ΔVpaChn25_0714, and ΔVpaChn25_RS25055-0713-0714 Mutants and Host Intestinal Epithelial Cells

**Figure 6:**
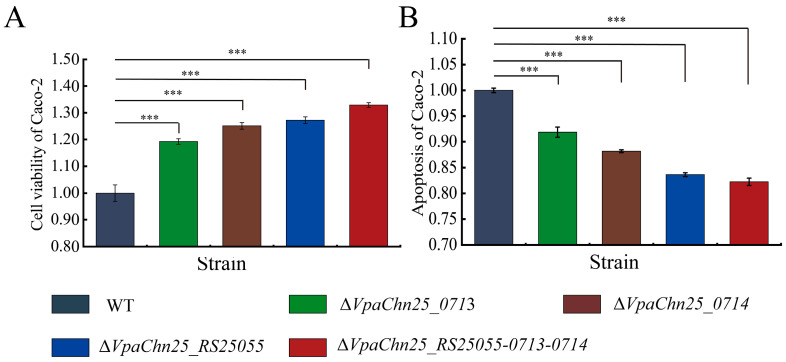
The viability and apoptosis of Caco-2 cells infected by the *V. parahaemolyticus* CHN25 WT, Δ*VpaChn25_RS25055*, Δ*VpaChn25_0713*, Δ*VpaChn25_0714*, and Δ*VpaChn25_RS25055-0713-0714* strains. (**A**) Cell viability; (**B**) cell apoptosis. *** *p* < 0.01.

In this study, Caco-2 was employed as a cell model for in vitro cell interaction assessment, and the data are presented in Figure 6 and Figure S14. Following infection with the Δ*VpaChn25_RS25055*, Δ*VpaChn25_0713*, Δ*VpaChn25_0714*, and Δ*VpaChn25_RS25055-0713-0714* mutants at 37 °C for 4 h, the survival of Caco-2 cells was remarkably increased by 1.27-fold, 1.19-fold, 1.23-fold, and 1.33-fold, respectively, as compared to the WT strain (*p* < 0.01) (Figure 6A). Concurrently, Caco-2 cells were subjected to double staining with the membrane-linked protein V-PI and FITC, followed by a flow cytometry assay analysis. It was found that the Δ*VpaChn25_RS25055*, Δ*VpaChn25_0713*, Δ*VpaChn25_0714*, and Δ*VpaChn25_RS25055-0713-0714* mutants induced Caco-2 cell apoptosis at 0.85-fold, 0.92-fold, 0.88-fold, and 0.83-fold lower rates than that of the WT strain following 4 h of infection, respectively (*p* < 0.01) (Figure 6B). This indicates that the deletion of the *VpaChn25_0713*, *VpaChn25_0714*, and *VpaChn25_RS25055* genes could reduce the ability of *V. parahaemolyticus* CHN25 to infect and apoptose the host intestinal epithelial Caco-2 cells.

### 2.8. The Major Changed Metabolic Pathways in the ΔVpaChn25_RS25055, ΔVpaChn25_0713, ΔVpaChn25_0714, and ΔVpaChn25_RS25055-0713-0714 Mutants

To assess the global-level expression alterations regulated by *VpaChn25_RS25055*, *VpaChn25_0713*, *VpaChn25_0714*, and *VpaChn25_RS25055-0713-0714* gene deletion, we determined the transcriptomes of the WT, the four mutants (Δ*VpaChn25_0713*, Δ*VpaChn25_RS25055*, Δ*VpaChn25_0714*, Δ*VpaChn25_RS25055-0713-0714*), and the four complementary mutants (Δ*VpaChn25_0713-*com, Δ*VpaChn25_RS25055-*com, Δ*VpaChn25_0714-*com, Δ*VpaChn25_RS25055-0713-0714-*com) cultivated in TSB medium to mid-logarithmic growth phase (mid-LGP) at 37 °C. The DEGs across all nine strains were deposited in the NCBI-SRA database (http://www.ncbi.nlm.nih.gov/sra/ (accessed on 27 February 2023); PRJNA938975). To validate the transcriptome data, we detected six representative genes in the Δ*VpaChn25_RS25055*, Δ*VpaChn25_0713*, Δ*VpaChn25_0714*, and Δ*VpaChn25_RS25055-0713-0714* mutants by RT-qPCR analysis, respectively (Table S1). The resulting data were correlated with those derived from transcriptome analyses (Table S2).

#### 2.8.1. The Major Changed Metabolic Pathways in the Δ*VpaChn25_0713* Mutant

**Figure 7:**
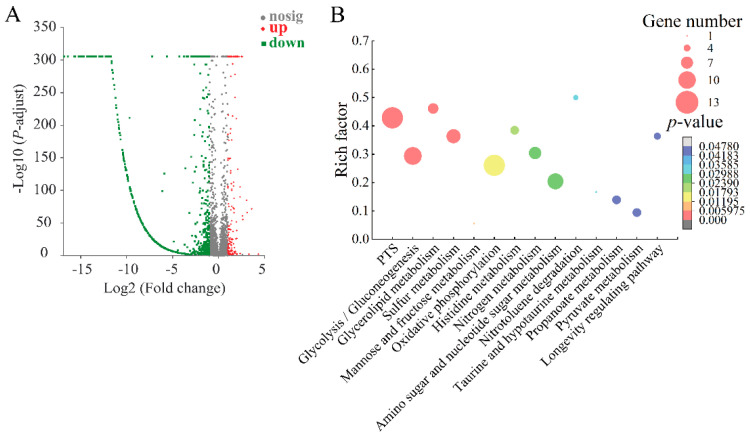
The volcano plot of differential gene expression (**A**) and the major changed metabolic pathways (**B**) in Δ*VpaChn25_0713*.

In the Δ*VpaChn25_0713* mutant, differential expression changes were observed in approximately 17.32% (812/4810) of the bacterial genes compared to both the WT and Δ*VpaChn25_0713-com* strains. According to KEGG database analysis of transcriptomic data, 14 significantly altered metabolic pathways were detected: the phosphotransferase system (PTS), glycolysis/gluconeogenesis, glycerolipid metabolism, mannose and fructose metabolism, sulfur metabolism, oxidative phosphorylation, histidine metabolism, nitrogen metabolism, amino sugar and nucleotide sugar metabolism, nitrotoluene degradation, taurine and hypotaurine metabolism, propanoate metabolism, longevity-regulating pathway, and pyruvate metabolism (Figure 7, Table S3).

For example, in the PTS, nine DEGs were markedly down-regulated at the mRNA level (0.057-fold to 0.491-fold) (*p* < 0.05). The PTS performs dual roles, facilitating the transport and phosphorylation of various sugars and their derivatives while also serving as a regulatory hub governing carbon, nitrogen, and phosphate metabolism, chemotaxis, potassium transport, and influencing the virulence of specific pathogens [32]. The significantly down-regulated DEGs may be consistent with the growth and biofilm-deficient phenotype of the Δ*VpaChn25_0713* mutant.

In glycolysis/gluconeogenesis, seven DEGs were remarkably decreased (0.203-fold to 0.494-fold) (*p* < 0.05): the type I glyceraldehyde-3-phosphate dehydrogenase (*Vpachn25_RS10585*), D-hexose-6-phosphate mutarotase (*VpaChn25_RS10590*), bifunctional acetaldehyde-CoA/alcohol dehydrogenase (*VpaChn25_RS10475*), pyruvate kinase (*VpaChn25_RS19575*), 6-phospho-*β*-glucosidase (*VpaChn25_RS23575*), triose-phosphate isomerase (*VpaChn25_RS01325*), and phosphoenolpyruvate synthase (*VpaChn25_RS17630*). The down-regulated DEGs associated with glycolysis/gluconeogenesis could lead to a polysaccharide deficiency. This deficiency might contribute to the biofilm formation observed in the Δ*VpaChn25_0713* mutant [33].

All five DEGs were markedly down-regulated in the pyruvate metabolism (0.162-fold to 0.421-fold) (*p* < 0.05), such as phosphoenolpyruvate carboxylase (*VpaChn25_RS14110*, 0.342-fold) (*p* < 0.05), which catalyzes the irreversible reaction between phosphoenolpyruvate (PEP) and bicarbonate to form inorganic phosphate and oxaloacetate, an essential step in bacteria and plants [34]. Four DEGs were decreased in amino sugar and nucleotide sugar metabolism (0.001-fold to 0.164-fold) (*p* < 0.05). Among these, the DEG encoding the UDP-glucose 4-epimerase GalE (*VpaChn25_RS21110*) was highly inhibited (0.001-fold). It facilitates the NAD-dependent interconversion of galacto- and gluco-hexoses, which is linked to UDP and holds a crucial position in the galactose metabolism of diverse organisms [35]. These two metabolic pathways are linked to carbohydrate metabolism, which is essential for all life and has implications for organisms’ growth, reproduction, and maintenance [36].

Remarkably, a total of 27 DEGs involved in energy metabolism were significantly changed, including nitrogen metabolism, sulfur metabolism, and oxidative phosphorylation, with 17 DEGs showing down-regulation (0.096-fold to 0.499-fold) (*p* < 0.05) and 10 genes showing higher transcript levels (2.081-fold to 4.053-fold) (*p* < 0.05). For example, in nitrogen metabolism, the DEG encoding a glutamate synthase subunit *β* (GltS *β* subunit) (*VpaChn25_RS02355*) was highly inhibited (0.096-fold), which is a flavin adenosine dinucleotide (FAD)-dependent nicotinamide adenine dinucleotide phosphate (NADPH) oxidoreductase, and serves to input electrons into the GltS α subunit for glutamate synthesis [37]. The expression of ATP binding cassette (ABC) transporter ATP-binding protein (*VpaChn25_RS20680*) was significantly decreased (0.231-fold); ABC proteins transport a huge range of diverse substrates, from simple ions through molecules to peptides, complex lipids, and even small proteins [38]. Moreover, in the sulfur metabolism, the DEG encoding CysK (*VpaChn25_RS04200*) was significantly inhibited (0.498-fold) (*p* < 0.05). Singh et al. showed that CysK is a key enzyme in the cysteine biosynthetic pathway involved in promoting biofilm formation [39]. These data suggested inactive transport and utilization of the carbon sources and repressed energy production in the Δ*VpaChn25_0713* mutant.

Comparative transcriptome analysis also showed the remarkably up-regulated metabolic routes (*p* < 0.05) in the Δ*VpaChn25_0713* mutant, such as histidine metabolism and propanoate metabolism. For example, four DEGs for histidine metabolism were remarkably increased (3.843-fold to 4.716-fold) (*p* < 0.05). Of these, imidazolonepropionase (*VpaChn25_RS06780*), which catalyzes histidine degradation, was substantially up-regulated (4.142-fold) (*p* < 0.05), while it mediates the third stage in the histidine degradation pathway. This enzyme hydrolyzes the carbon- nitrogen bonds within 4-imidazolone-5-propionic acid, forming N-formimino-l-glutamic acid [40]. The substantial elevation of these enzymes indicates that deletion of the *VpaChn25_0713* gene affects histidine metabolism and may promote histidine degradation.

Moreover, all six DEGs involved in glycerolipid metabolism were significantly up-regulated (*p* < 0.05). Glycerolipids are a class of biological molecules required for membrane formation, caloric storage, and important intracellular signaling processes [41]. The overall up-regulation of the glycerolipid metabolism provides new insight into the mechanism by which *VpaChn25_0713* gene deletion in *V. parahaemolyticus* may affect lipid metabolism. In contrast, nitrotoluene degradation involved three genes that underwent a significant decrease (*p* < 0.05). Nitroreductases in the intestinal microbiota are involved in the biotransformation of several poisonous, mutagenic, and carcinogenic nitroaromatic chemicals’ reduction products to their hazardous metabolites [42]. Therefore, it is hypothesized that changes in this metabolic pathway correlate with the relevant results of cytotoxicity assays.

Taken together, these data suggested that *VpaChn25_0713* gene deletion could inhibit the transportation and phosphorylation of sugar compounds and their derivatives, and suppress the glycolytic/glucose metabolic pathway, leading to polysaccharide deficiency; it inhibited the production of glutamate as well as cysteine, thus affecting energy production. The above changes may contribute to the reduced swimming ability, biofilm formation, and decreased virulence of the Δ*VpaChn25_0713* mutant.

#### 2.8.2. The Major Changed Metabolic Pathways in the Δ*VpaChn25_0714* Mutant

**Figure 8:**
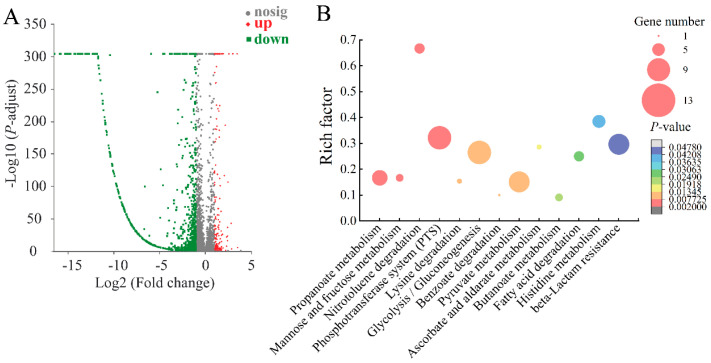
The volcano plot of differential gene expression (**A**) and the major changed metabolic pathways (**B**) in Δ*VpaChn25_0714.*

In the Δ*VpaChn25_0714* mutant, differential expression changes were observed in approximately 19.17% (922/4810) of the bacterial genes compared to both the WT and Δ*VpaChn25_0714-com* strains. According to KEGG database analysis of transcriptomic data, 13 significantly altered metabolic pathways were detected: the propanoate metabolism, mannose and fructose metabolism, nitrotoluene degradation, PTS, lysine degradation, glycolysis/gluconeogenesis, benzoate degradation, pyruvate metabolism, ascorbate and aldarate metabolism, butanoate metabolism, fatty acid degradation, histidine metabolism, and *β*-lactam resistance (Figure 8, Table S4).

Similar to the Δ*VpaChn25_0713* mutant, most DEGs related to mannose and fructose metabolism, nitrotoluene degradation, PTS, glycolysis/gluconeogenesis, and pyruvate metabolism were also markedly reduced in Δ*VpaChn25_0714*. For example, two DEGs were down-regulated at the mRNA level in mannose and fructose metabolism (0.08-fold to 0.426-fold) (*p* < 0.05). Apart from being an energy and carbon source, fructose metabolism has been shown to impact various cellular processes, including biofilm formation in streptococci and the pathogenicity of bacteria in plants [43].

In addition, 22 DEGs were significantly down-regulated (0.080-fold to 0.494-fold) (*p* < 0.05), and 9 DEGs were significantly up-regulated (2.017-fold to 3.217-fold) (*p* < 0.05) in carbohydrate metabolism. They involved a total of six types of metabolic pathways, which were propanoate metabolism, mannose and fructose metabolism, glycolysis/gluconeogenesis metabolism, pyruvate metabolism, ascorbate and aldarate metabolism, and butanoate metabolism. Overall, these metabolic pathways showed an overall trend of down-regulation. For example, in propanoate metabolism, five DEGs underwent significant down-regulation (0.233-fold to 0.435-fold) (*p* < 0.05). Of these, acetate kinase is an enzyme widely distributed in the bacteria and archaea domains which catalyzes the phosphorylation of acetate [44]. In glycolysis/gluconeogenesis metabolism, six DEGs underwent significant down-regulation (0.228-fold to 0.483-fold) (*p* < 0.05). Of these, the DEG encoding type I glyceraldehyde-3-phosphate dehydrogenase (*VpaChn25_RS10585*, 0.285-fold) was significantly down-regulated, which is essential for glycolysis [45]. The down-regulated DEGs involved in carbohydrate metabolism may be responsible for the biofilm formation defect in the Δ*VpaChn25_0714* mutant.

In *β*-lactam resistance, all eight DEGs were remarkably decreased (0.119-fold to 0.462-fold) in the Δ*VpaChn25_0714* mutant (*p* < 0.05). Resistance is frequently acquired through the action of *β*-lactamases or the expression of alternative *β*-lactam-resistant penicillin-binding proteins (PBPs) [46].

Comparative transcriptome analysis indicated that several metabolic routes were markedly elevated in Δ*VpaChn25_0714* (*p* < 0.05). For example, in histidine metabolism, four DEGs were markedly increased (3.400-fold to 4.629-fold) (*p* < 0.05): histidine ammonia-lyase (*VpaChn25_RS06765*), imidazolonepropionase (*VpaChn25_RS06780*), urocanate hydratase (*VpaChn25_RS06770*), and formimidoylglutamase (*VpaChn25_RS06775*). Of these, the DEG encoding histidine ammonia-lyase (*VpaChn25_RS06765*, 3.4-fold) regulates the initial step in histidine catabolism by catalyzing the deamination of histidine into urocanate and ammonia [47]. Meanwhile, in fatty acid degradation, three DEGs were markedly elevated (2.100-fold to 2.425-fold) (*p* < 0.05), including the fatty acid oxidation complex subunit *α* FadJ (*VpaChn25_RS10820*), acetyl-CoA C-acyltransferase FadI (*VpaChn25_RS10825*), and acetyl-CoA C-acetyltransferase (*VpaChn25_RS18175*). Fatty acids play an important role in the structural composition of cellular membranes and serve various functions in biological processes [48].

Taken together, similar to the Δ*VpaChn25_0713* mutant, the deletion of the *VpaChn25_0714* gene also inhibits the PTS pathway, promoting fatty acid degradation and suppressing the glycolytic/glucose metabolic pathway, leading to polysaccharide deficiency. The above changes may be related to the growth and biofilm-deficient phenotype of the Δ*VpaChn25_0714* mutant.

#### 2.8.3. The Major Changed Metabolic Pathways in the Δ*VpaChn25_RS25055* Mutant

**Figure 9:**
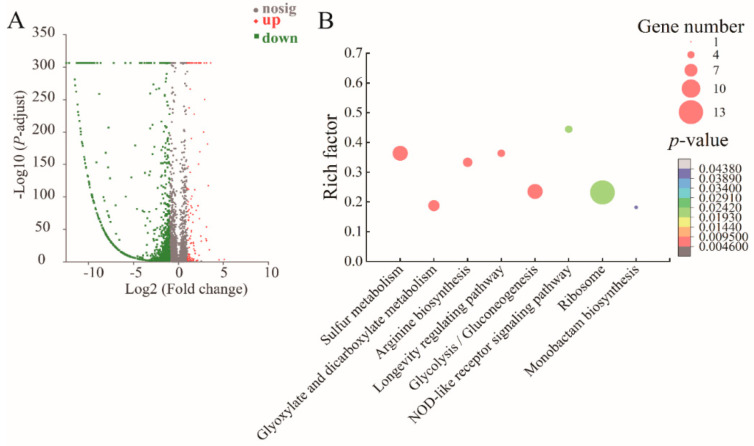
The volcano plot of differential gene expression (**A**), and the major changed metabolic pathways (**B**) in Δ*VpaChn25_RS25055.*

In the Δ*VpaChn25_RS25055* mutant, there were differential expression changes observed in approximately 16.59% (798/4810) of the bacterial genes when compared to both the WT and Δ*VpaChn25_RS25055-com* strains. According to the KEGG database analysis of transcriptomic data, eight significantly altered metabolic pathways were detected: sulfur metabolism, glyoxylate and dicarboxylate metabolism, arginine biosynthesis, longevity-regulating pathway, glycolysis/gluconeogenesis, NOD-like receptor signaling pathway, ribosome, and monobactam biosynthesis (Figure 9, Table S5).

Similar to the Δ*VpaChn25_0713* mutant, most DEGs in the sulfur metabolism, glycolysis/gluconeogenesis, and longevity-regulating pathways were also remarkably reduced in the Δ*VpaChn25_RS25055* mutant.

In addition, all five DEGs were significantly down-regulated (0.084-fold to 0.305-fold) (*p* < 0.05) in arginine biosynthesis. Among them, N-acetylglutamate kinase (NAGK) catalyzes the second step of arginine biosynthesis [49]. Similarly, argininosuccinate synthetase 1 (ASS1) is a rate-limiting enzyme in arginine biosynthesis [50]. Overall, *E. coli* uses arginine as its only nitrogen supply, and many other bacteria use it as a source of nitrogen, carbon, and energy [51]. It has been shown that intracellular arginine deficiency may affect the formation of biofilms [52,53].

In the NOD-like receptor signaling pathway, all four DEGs were significantly down-regulated (0.317-fold to 0.451-fold) (*p* < 0.05), including flagellin (*VpaChn25_RS04175*, *VpaChn25_RS11070*, *VpaChn25_RS11075*) and the molecular chaperone HtpG (*VpaChn25_RS04340*). Flagellin, which polymerizes into flagellar filament, is essential for bacterial motility, and flagella-driven motility is an important trait of bacterial colonization and virulence [54,55].

Comparative transcriptome analysis demonstrated that several metabolic routes were remarkably up-regulated (*p* < 0.05) in Δ*VpaChn25_RS25055*, including ribosome, glyoxylate, and dicarboxylate metabolism. Briefly, 18 DEGs had significantly up-regulated transcript levels (2.006-fold to 6.026-fold) (*p* < 0.05). In the ribosome, all 13 DEGs were increased (2.006-fold to 2.705-fold) (*p* < 0.05). Ribosomes are macromolecular complexes in the cytoplasm, consisting of proteins and RNA, which connect amino acids and synthesize new proteins [56]. For example, the DEG (*VpaChn25_RS02135*) encoding a 50S ribosomal protein L13 was up-regulated (2.191-fold) (*p* < 0.05). Aseev et al. revealed that L13 was a major protein in the assembly of the 50S ribosomal subunit and serves as a repressor of rplM-rpsI expression in vivo. [57]. This indicated dramatic cellular reprogramming in the Δ*VpaChn25_RS25055* mutant [58]. 

Taken together, this indicates that the deletion of the *VpaChn25_RS25055* gene inhibits the transport and utilization of carbon sources, inhibits the biosynthesis of arginine and the formation of flagella, and changes the biosynthesis of ribosomes. These DEGs induced by *VpaChn25_RS25055* deletion may affect swimming and virulence.

#### 2.8.4. The Major Changed Metabolic Pathways in the Δ*VpaChn25_RS25055-0713-0714* Mutant

**Figure 10:**
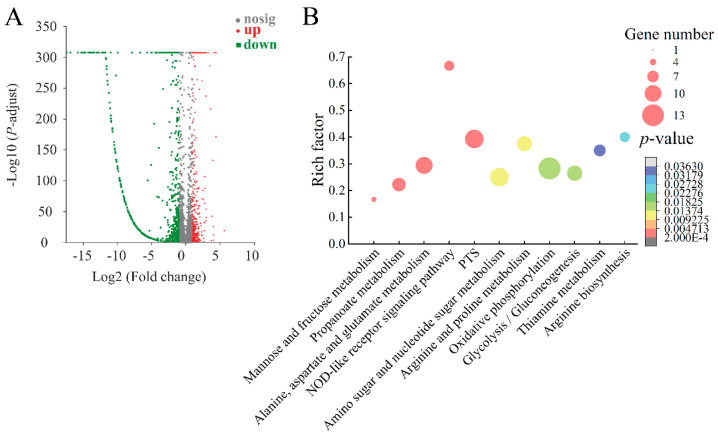
The volcano plot of differential gene expression (**A**), and the major changed metabolic pathways (**B**) in the Δ*VpaChn25_RS25055-0713-0714* mutant.

In the Δ*VpaChn25_RS25055-0713-0714* mutant, there were differential expression changes observed in approximately 19.90% (957/4810) of the bacterial genes when compared to both the WT and Δ*VpaChn25_RS25055-0713-0714-com* strains. According to KEGG database analysis of transcriptomic data, 11 significantly altered metabolic pathways were detected, such as the mannose and fructose metabolism, propanoate metabolism, alanine, aspartate and glutamate metabolism, NOD-like receptor signaling pathway, PTS, amino sugar and nucleotide sugar metabolism, arginine and proline metabolism, oxidative phosphorylation, glycolysis/gluconeogenesis, thiamine metabolism, and arginine biosynthesis (Figure 10, Table S6).

Similar to the Δ*VpaChn25_0713* mutant, most DEGs related to mannose and fructose metabolism, glycolysis/gluconeogenesis, and PTS were decreased in the Δ*VpaChn25_RS25055-0713-0714* mutant. Meanwhile, most DEGs related to NOD-like receptor signal transduction and arginine biosynthesis were also markedly reduced in the Δ*VpaChn25_RS25055-0713-0714* mutant, similar to the Δ*VpaChn25_RS25055* mutant.

The major metabolic pathway altered by the triple-gene-deletion mutant (Δ*VpaChn25_RS25055-0713-0714*) was not identical to that altered by the single-gene mutants (Δ*VpaChn25_RS25055*, Δ*VpaChn25_0713*, Δ*VpaChn25_0714*). Comparative transcriptomics revealed that the metabolic pathway of glycolysis/gluconeogenesis exhibited an overall down-regulation in the deletion of the single genes *VpaChn25_RS25055*, *VpaChn25_0713*, and *VpaChn25_0714*, while the deletion of three genes (*VpaChn25_RS25055-0713-0714*) showed an overall down-regulated superposition effect. In addition, significant changes in mannose and fructose metabolism, propanoate metabolism, and amino sugar and sugar metabolism were simultaneously observed in the four deletion mutants in this study. Carbohydrates can be catabolized for energy (ATP) or employed for anabolic functions [59]. Combining the experimental data on growth, swimming, and biofilm-related phenotypes mentioned in the previous section, it is hypothesized that the genes *VpaChn25_RS25055*, *VpaChn25_0713*, and *VpaChn25_0714* are synergistically involved in regulating the active transport and utilization of carbon sources.

In the NOD-like receptor signaling pathway, all six DEGs were remarkably decreased (0.331-fold to 0.425-fold) (*p* < 0.05). Bacterial polar flagella, containing flagellin, play a vital role in bacterial motility. Swimming motility is an essential virulence factor for the pathogenesis of many *Vibrio* species [60,61]. It has been reported that in gut inflammation, *Clostridioides difficile* flagellin FliC plays a role in toxin contribution by interacting with the TLR5 of the immune system, triggering the activation of NF-kB and MAPK signal transduction [62]. The data indicate a potential issue with the flagellar basal body in the Δ*VpaChn25_RS25055-0713-0714* mutant, which could have affected its impaired swimming ability and reduced virulence.

In the PTS, eight DEGs were transcriptionally significantly repressed (0.056-fold to 0.405-fold) (*p* < 0.05). Comparative transcriptomics revealed that PTS showed an overall down-regulation in the deletion of single genes *VpaChn25_0713* and *VpaChn25_0714*, while the deletion of three genes (*VpaChn25_RS25055-0713-0714*) showed a superimposed effect of multiple genes acting together. For example, the Δ*VpaChn25_RS25055-0713-0714* mutant encoding fused PTS fructose transporter subunit IIA/HPr (*VpaChn25_RS19530*, 0.122-fold) showed lower expression than Δ*VpaChn25_0713* (0.136-fold) and Δ*VpaChn25_0714* (0.134-fold). The PTS mediates both the uptake of carbohydrates across the cytoplasmic membrane and their phosphorylation [63]. Combined with the experimental data of biofilm-related phenotypes, we believe that *VpaChn25_0713* and *VpaChn25_0714* synergistically regulate membrane transport.

In addition, six DEGs were significantly down-regulated in alanine, aspartate, and glutamate metabolism (0.098-fold to 0.480-fold) (*p* < 0.05). Meanwhile, in arginine biosynthesis, all six DEGs were significantly repressed (0.101-fold to 0.376-fold) (*p* < 0.05). This may help *V. parahaemolyticus* maintain the stability of bacterial cell structure and function.

Comparative transcriptome analysis also revealed the significantly up-regulated metabolic pathways (*p* < 0.05) in the Δ*VpaChn25_RS25055-0713-0714* mutant. For example, all DEGs were significantly up-regulated in oxidative phosphorylation (2.218-fold to 5.684-fold) (*p* < 0.05), a metabolic pathway related to energy metabolism; all DEGs were significantly up-regulated in thiamine metabolism, a metabolic pathway related to the metabolism of cofactors and vitamins; in arginine and proline metabolism, six DEGs were significantly up-regulated (2.105-fold to 3.110-fold) (*p* < 0.05).

Taken together, it is indicated that the deletion of the *VpaChn25_RS25055-0713-0714* gene inhibits the transport and utilization of carbon sources, the formation of flagella, and the biosynthesis of glutamate and arginine. The above changes may also contribute to the delayed growth, reduced swimming ability, biofilm formation, and decreased virulence of the Δ*VpaChn25_RS25055-0713-0714* mutant.

#### 2.8.5. Possible Molecular Mechanisms of the Δ*VpaChn25_RS25055*, Δ*VpaChn25_0713*, Δ*VpaChn25_0714*, and Δ*VpaChn25_RS25055-0713-0714* Mutants

**Figure 11:**
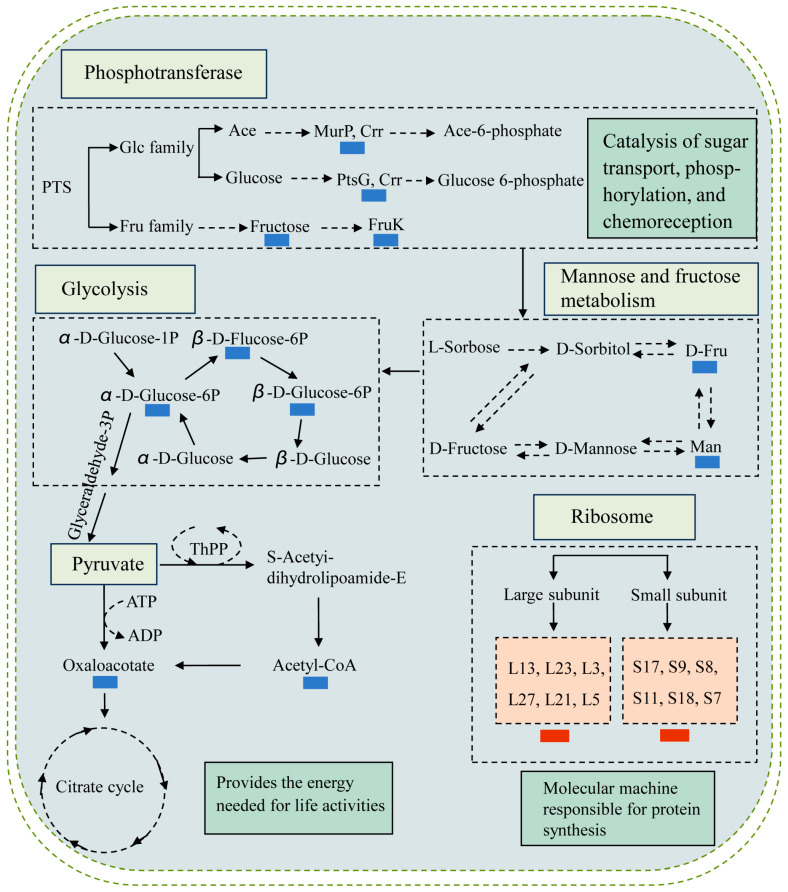
Possible molecular mechanisms in Δ*VpaChn25_RS25055*, Δ*VpaChn25_0713*, Δ*VpaChn25_0714*, and Δ*VpaChn25_RS25055-0713-0714* mutants. Red boxes indicate an increased abundance of genes or proteins, while blue boxes indicate reduced abundance of genes or proteins. Ace: N-Acetylmuramic acid; Ace-6-phosphate: N-Acetylmuramic acid 6-phosphate; Man: D-Mannose-6 P; Fru: *β*-D-Fructose-6 P; D-Fru: *β*-D-Fructose-1, 6 P_2_.

In the present study, the transcriptome analyses revealed several DEGs involved in multiple pathways of biosynthesis, degradation, interconversion, and transport of the compounds in the Δ*VpaChn25_RS25055*, Δ*VpaChn25_0713*, Δ*VpaChn25_0714*, and Δ*VpaChn25_RS25055-0713-0714* mutants, indicating a complex molecular regulation network in the absence of the above prophage genes (Figure 11).

The same metabolic pathways were elicited in the deletion mutants. For instance, the repressed glycolysis/gluconeogenesis metabolism in the Δ*VpaChn25_RS25055*, Δ*VpaChn25_0713*, Δ*VpaChn25_0714*, and Δ*VpaChn25_RS25055-0713-0714* mutants; the repressed PTS, mannose, and fructose metabolism in the Δ*VpaChn25_0713*, Δ*VpaChn25_0714*, and Δ*VpaChn25_RS25055-0713-0714* mutants; and the repressed pyruvate metabolism in the Δ*VpaChn25_0713* and Δ*VpaChn25_0714* mutants.

We also observed different metabolic pathways occurring in the above mutants. For instance, in the Δ*VpaChn25_RS25055* mutant, 13 DEGs in the ribosome were markedly increased (2.006-fold to 2.705-fold) (*p* < 0.05).

Overall, multiple metabolic pathways were changed in the above mutants: (1) the PTS was down-regulated, which affects sugar transport, phosphorylation, and chemoreception; (2) the mannose and fructose metabolism, glycolysis, and pyruvate metabolism were down-regulated, thereby affecting energy production; and (3) the amino acid synthesis was decreased to delay cell growth.

### 2.9. SEM Observation of Cell Structure of the ΔVpaChn25_RS25055, ΔVpaChn25_0713, ΔVpaChn25_0714, and ΔVpaChn25_RS25055-0713-0714 Mutants

**Figure 12:**
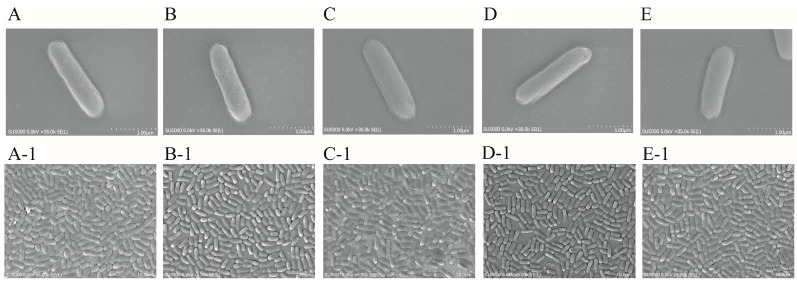
The SEM observation of the cell structure of the *V. parahaemolyticus* CHN25 (WT), Δ*VpaChn25_RS25055*, Δ*VpaChn25_0713*, Δ*VpaChn25_0714*, and Δ*VpaChn25_RS25055-0713-0714* strains. (**A**,**A-1**): WT; (**B**,**B-1**): Δ*VpaChn25_0713*; (**C**,**C-1**): Δ*VpaChn25_0714*; (**D**,**D-1**): Δ*VpaChn25_RS25055*; (**E**,**E-1**): Δ*VpaChn25_RS25055-0713-0714*.

The bacterial cell structures of the *V. parahaemolyticus* CHN25 WT, four mutants, and four complementary mutants were evaluated by SEM analysis (Figure 12 and Figure S15). As shown in Figure 12, all strains have intact cell surface structures, showing rod-shaped cells with a flat surface in the TSB medium (3% NaCl, pH 8.5) at 37 °C.

### 2.10. Distribution of the VpaChn25_RS25055, VpaChn25_0713, and VpaChn25_0714 Genes in Bacteria

A total of 119 *V. parahaemolyticus* isolates, which were recovered from aquatic products collected in Shanghai, China [64], were tested for the *VpaChn25_RS25055*, *VpaChn25_0713*, and *VpaChn25_0714* genes via the PCR assays. The findings indicated that 1.68% (*n* = 1) of the isolates harbored *VpaChn25_RS25055*, *VpaChn25_0713*, and *VpaChn25_0714* homologs, respectively.

**Figure 13:**
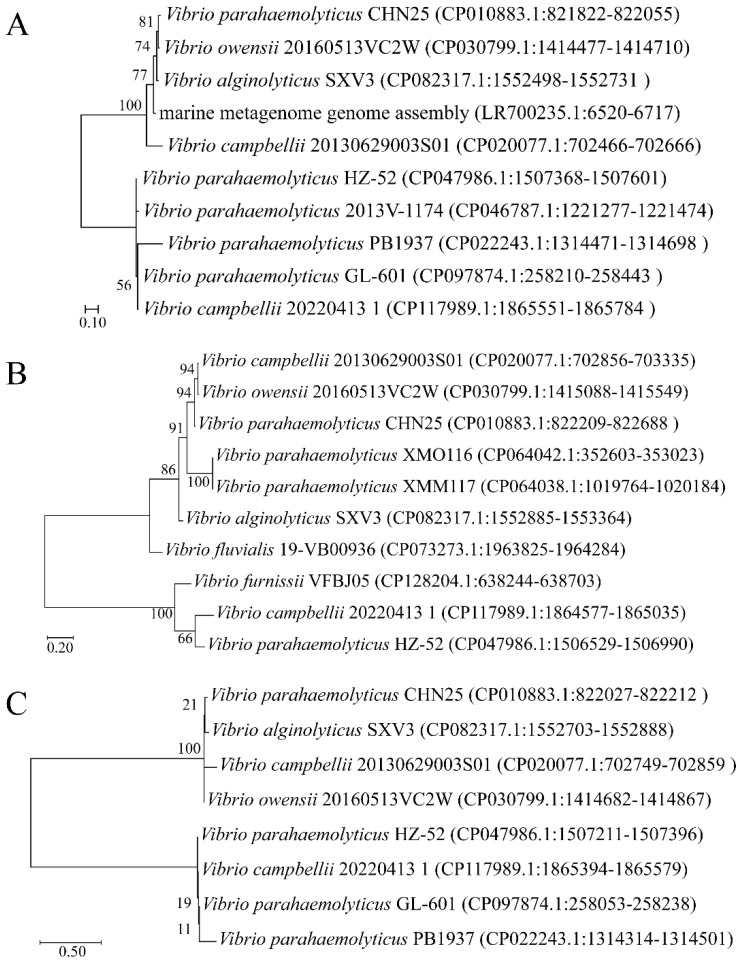
Phylogenetic relationships between the *VpaChn25_RS25055*, *VpaChn25_0713*, and *VpaChn25_0714* genes and their homologs. *VpaChn25_0713* phylogenetic tree (**A**), *VpaChn25_0714* phylogenetic tree (**B**), *VpaChn25_RS25055* phylogenetic tree (**C**).

Analysis using BLAST against the GenBank database demonstrated the presence of the homologs of *VpaChn25_0713* in a single *Vibrio* phage, vB_VpaM_VP-3212, which is part of the marine metagenome genome assembly LR700235; and eight *Vibrio* species, including *Vibrio owensii* 20160513VC2W (CP030799), *Vibrio alginolyticus* SXV3 (CP082317), *Vibrio campbellii* 20130629003S01 (CP020077), and *V. parahaemolyticus* 2013V-1174 (CP046787), etc. (Figure 13A).

BLAST analysis against the GenBank database showed that *VpaChn25_0714* homologs were present in nine *Vibrio* species, including *V. campbellii* 20130629003S01 (Genbank accession no.: CP020077), *V. owensii* 20160513VC2W (Genbank accession no.: CP030799), *V. alginolyticus* SXV3 (Genbank accession no.: CP082317), *V. parahaemolyticus* XMO116 (Genbank accession no.: CP064042), and *Vibrio fluvialis* 19-VB00936 (Genbank accession no.: CP073273), etc. (Figure 13B).

BLAST analysis against the GenBank database also revealed that *VpaChn25_RS25055* homologs were present in seven *Vibrio* species, including *V. owensii* 20160513VC2W (Genbank accession no.: CP030799), *V*. *alginolyticus* SXV3 (Genbank accession no.: CP082317), *V. parahaemolyticus* PB1937 (Genbank accession no.: CP022243), and *V. campbellii* 20130629003S01 (Genbank accession no.: CP020077), etc. (Figure 13C).

It was observed that the *VpaChn25_RS25055*, *VpaChn25_0713*, and *VpaChn25_0714* genes exist in *V. parahaemolyticus* and the *Vibrio* genus. Notably, the homologs of the genes *VpaChn25_RS25055*, *VpaChn25_0713*, and *VpaChn25_0714* are all present in *V. campbellii* 20130629003S01. *V*. *campbellii* is a crucial aquatic pathogen, capable of causing vibriosis in shrimp and fish, leading to substantial economic losses [65].

### 2.11. Cellular Localization of VpaChn25_RS25055 in V. parahaemolyticus CHN25

sfGFP has a stable β-barrel structure and superior features among GFP mutants, such as high solubility, bright fluorescence, fast folding ability, and high denaturant resistance [66]. Δ*VpaChn25_RS25055* (pMMB207+*VpaChn25_RS25055*-sfGFP) was successfully constructed by transferring *VpaChn25_RS25055*-sfGFP into Δ*VpaChn25_RS25055* to study the localization of *VpaChn25_RS25055* in cells (Figure S16). Meanwhile, Δ*VpaChn25_RS25055* (pMMB207+sfGFP) and Δ*VpaChn25_RS25055* (pMMB207) were constructed as controls. Sequencing results of Δ*VpaChn25_RS25055* (pMMB207), Δ*VpaChn25_RS25055* (pMMB207+sfGFP), and Δ*VpaChn25_RS25055* (pMMB207+*VpaChn25_RS25055*-sfGFP) are presented in Figures S17–S19.

**Figure 14:**
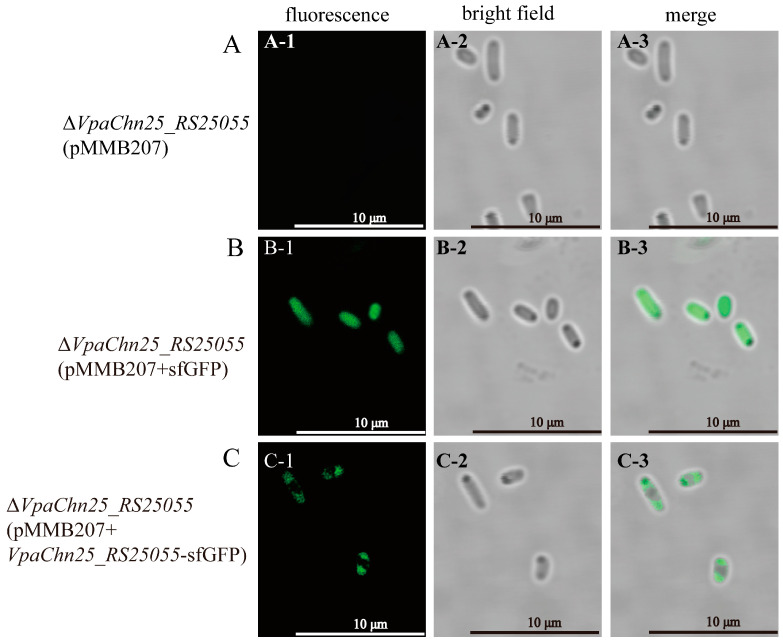
Localization of *VpaChn25_RS25055* in *V. parahaemolyticus* CHN25 under confocal microscope. (**A**) Negative control Δ*VpaChn25_RS25055* (pMMB207) strain is non-fluorescent under fluorescence (**A-1**) and bright field (**A-2**); (**B**) negative control Δ*VpaChn25_RS25055* (pMMB207+sfGFP) strain shows sfGFP distribution throughout the cytoplasm; (**C**) Δ*VpaChn25_RS25055* (pMMB207+*VpaChn25_RS25055*-sfGFP) strain under fluorescence (**C-1**) and bright field (**C-2**) showing that the *VpaChn25_RS25055*-sfGFP fusion protein is located at both cell poles.

The Δ*VpaChn25_RS25055* (pMMB207), Δ*VpaChn25_RS25055* (pMMB207+sfGFP), and Δ*VpaChn25_RS25055* (pMMB207+*VpaChn25_RS25055*-sfGFP) strains cultured in the TSB medium (pH 8.5, 3% NaCl) at 37 °C to mid-LGP were observed by a High-Resolution Laser Confocal Microscope. Figure 14 indicates no fluorescence in the negative control of Δ*VpaChn25_RS25055* (pMMB207) (A-1, A-2, and A-3 in Figure 14). Fluorescence in Δ*VpaChn25_RS25055* (pMMB207+sfGFP) was distributed in the cytoplasm (B-1, B-2, and and B-3 in Figure 14). However, the fluorescence of the fusion protein pMMB207+*VpaChn25_RS25055*-sfGFP was located at both poles of *V. parahaemolyticus* (C-1, C-2, and C-3 in Figure 14). This indicated that at this stage, the prophage gene *VpaChn25_RS25055* is localized at both cell poles.

## 3. Discussion

*V. parahaemolyticus* is a common foodborne pathogen capable of inducing acute gastroenteritis in humans [67]. The complete biological functions of the prophage-associated gene found in *V. parahaemolyticus* have yet to be comprehensively elucidated. In this study, the *VpaChn25_RS25055*, *VpaChn25_0713*, and *VpaChn25_0714* genes, which encoded hypothetical proteins in the *V. parahaemolyticus* CHN25 genome, were systematically studied for the first time. We successfully constructed the deletion mutants and complementary mutants. Our data indicated that the deletion of *VpaChn25_RS25055*, *VpaChn25_0713*, *VpaChn25_0714*, and *VpaChn25_RS25055-0713-0714* genes resulted in a defect in the growth of *V. parahaemolyticus* CHN25 at lower temperatures. In addition, the Δ*VpaChn25_RS25055-0713-0714* mutant, deleted with three genes, had a more extended lag phase at pH 5.5–8.0 than the WT strain and other mutants.

Flagella are organelles of locomotion that play an essential role in attachment, biofilm formation, and pathogenesis [68]. In numerous bacterial species, the ability for swimming motility is essential for their interactions with hosts [60]. In this study, our results indicated that the swimming motility of Δ*VpaChn25_RS25055*, Δ*VpaChn25_0713*, and Δ*VpaChn25_RS25055-0713-0714* mutants were significantly inhibited. Furthermore, the biofilm formation of *V. parahaemolyticus* CHN25 was reduced when *VpaChn25_RS25055*, *VpaChn25_0713*, *VpaChn25_0714*, and *VpaChn25_RS25055-0713-0714* genes were absent.

Traditionally, membrane fluidity has been regarded as a fundamental physical property influencing cell adhesion [69]. A noticeable increase in the membrane fluidity was seen in the Δ*VpaChn25_0713*, Δ*VpaChn25_0714*, and Δ*VpaChn25_RS25055* mutants compared to the WT (*p* < 0.01). As membrane fluidity increases, membrane permeability to water and other hydrophilic small molecules increases. Additionally, the hydrophobicity of bacterial cell surfaces is an important characteristic that plays a role in determining a bacterium’s capacity to adhere to non-reactive surfaces [70]. In this study, the Δ*VpaChn25_RS25055* and Δ*VpaChn25_RS25055-0713-0714* mutants underwent a significant decrease in cell surface hydrophobicity.

In the Caco-2 cell model in vitro, all four mutants significantly reduced the cytotoxicity of *V. parahaemolyticus* CHN25 to human intestinal epithelial cells (*p* < 0.01). Notably, the apoptosis rate of Caco-2 cells infected by Δ*VpaChn25_RS25055* and Δ*VpaChn25_RS25055-0713-0714* were significantly smaller than that of Δ*VpaChn25_0713* and Δ*VpaChn25_0714* mutants. This showed that expression of the *VpaChn25_RS25055*, *VpaChn25_0713*, and *VpaChn25_0714* genes benefited *V. parahaemolyticus* CHN25 for its infection of host cells.

Transcriptomic analysis showed that 15, 14, 8, and 11 metabolic pathways were significantly changed in the Δ*VpaChn25_RS25055*, Δ*VpaChn25_0713*, Δ*VpaChn25_0714*, and Δ*VpaChn25_RS25055-0713-0714* mutants, respectively. Moreover, they were mainly involved in membrane transport, carbohydrate metabolism, energy metabolism, translation, and other metabolic pathways. In summary, the prophage-encoding genes *VpaChn25_RS25055*, *VpaChn25_0713*, *VpaChn25_0714*, and *VpaChn25_RS25055-0713-0714* enhance the adaptability of *V. parahaemolyticus* CHN25 to survive in the environment and the host. The results of this study contribute to a better understanding of the pathogenicity and evolution of *V. parahaemolyticus*.

Remarkably, an overall down-regulation of glycolysis/gluconeogenesis metabolism occurred in the Δ*VpaChn25_RS25055*, Δ*VpaChn25_0713*, Δ*VpaChn25_0714*, and Δ*VpaChn25_RS25055-0713-0714* mutants. In particular, *VpaChn25_RS10585* (type I GAPDH), *VpaChn25_RS10590* (D-hexose-6-phosphate mutarotase), *VpaChn25_RS10475* (bifunctional acetaldehyde-CoA/alcohol dehydrogenase), and *VpaChn25_RS23575* (6-phospho-*β*-glucosidase) were significantly down-regulated in all deletion strains. Among these, GAPDH contributes to biological processes such as membrane fusion, DNA replication and repair, and apoptosis [45]. Recent studies have shown that type 1 GAPDH (Ec GAPDH1) from *E. coli* is responsible for protein synthesis, protein folding, and DNA repair [45]. The down-regulated DEGs associated with glycolysis/gluconeogenesis led to a deficiency in polysaccharide production, which, in turn, contributed to the biofilm formation defect observed in the deletion mutants [33].

Meanwhile, in the Δ*VpaChn25_0713* mutant, six DEGs were remarkably decreased in the pyruvate metabolism (0.162-fold to 0.421-fold) (*p* < 0.05). Four DEGs were markedly reduced in amino sugar and nucleotide sugar metabolism (0.001-fold to 0.164-fold) (*p* < 0.05). In the Δ*VpaChn25_0714* mutant, propanoate, fructose, mannose, and pyruvate metabolism underwent an overall down-regulation. Mannose and fructose metabolism were also obviously decreased in the Δ*VpaChn25_RS25055-0713-0714* mutant, which is all related to carbohydrate metabolism. These findings indicate a lack of active transportation and usage of carbon sources, along with suppressed energy production in Δ*VpaChn25_RS2505*5, Δ*VpaChn25_0713*, Δ*VpaChn25_0714*, and Δ*VpaChn25_RS25055-0713-0714* mutants.

PTS also serves as a sophisticated protein kinase system that governs a diverse range of metabolic processes, transport mechanisms, and the expression of numerous genes. It establishes a connection between the PTS and the virulence of specific pathogens [71,72]. In this study, the overall down-regulation of PTS metabolism occurred in the Δ*VpaChn25_0713*, Δ*VpaChn25_0714*, and Δ*VpaChn25_RS25055-0713-0714* mutants.

In addition, significant down-regulation of all DEGs in the NOD-like receptor signaling pathway occurred in the Δ*VpaChn25_RS25055* and Δ*VpaChn25_RS25055-0713-0714* mutants. Flagella are essential in attachment, biofilm formation, and pathogenesis [68]. Down-regulation of these genes in the Δ*VpaChn25_RS25055* and Δ*VpaChn25_RS25055-0713-0714* mutants may have contributed to their observed defects in swimming motility, biofilm formation, and cytotoxicity to the host cells.

Comparative transcriptome analysis also revealed that a few metabolic pathways were significantly up-regulated (*p* < 0.05) in the mutants. For example, in the Δ*VpaChn25_0713* mutant, the oxidative phosphorylation, histidine metabolism, and propanoate metabolism were up-regulated (*p* < 0.05). Four DEGs in histidine metabolism were significantly up-regulated (3.843-fold to 4.716-fold) (*p* < 0.05), such as the highly up-regulated gene, *VpaChn25_RS06770* (4.374-fold) (*p* < 0.05) encoding a urocanate hydratase, which is involved in the L-histidine catabolic pathway and plays a significant role in providing intermediates for the TCA cycle [73]. In the Δ*VpaChn25_0714* mutant, four DEGs in histidine metabolism (3.400-fold to 4.629-fold) (*p* < 0.05) and three DEGs in fatty acid decomposition (2.100-fold to 2.425-fold) (*p* < 0.05) were significantly up-regulated. Fatty acids are essential components of cell membranes and an important source of metabolic energy in all organisms [74,75]. Remarkably, in the Δ*VpaChn25_RS25055* mutant, 13 DEGs in the ribosome were markedly increased (2.006-fold to 2.705-fold) (*p* < 0.05). Ribosomes are large molecular complexes that translate the genetic code into functional proteins [76]. The biogenesis of ribosomes includes rDNA transcription, rRNA processing, and the assembly of ribosomal proteins with rRNA; ribosomal protein has been shown to affect the RNA-to-protein ratio, and is necessary for cell growth [56]. In the Δ*VpaChn25_RS25055-0713-0714* mutant, all DEGs in oxidative phosphorylation (2.218-fold to 5.684-fold) (*p* < 0.05) and thiamine metabolism (2.127-fold to 3.115-fold) (*p* < 0.05) were significantly up-regulated, which were related to energy metabolism and metabolism of cofactors and vitamins, respectively.

As was observed by fluorescence measurements, the fusion protein *VpaChn25_RS25055*-sfGFP is located at both poles of *V. parahaemolyticus* CHN25 during the mid-LGP stage. It has been reported that cell poles are specific assemblies of surface organelles such as flagella, pili, and virulence factor secretion systems, allowing the cell to orient itself for directional motility and interaction with surfaces [77]. It is also consistent with this study’s *V. parahaemolyticus* CHN25-deficient phenotype resulting from the *VpaChn25_RS25055* gene deletion.

## 4. Materials and Methods

### 4.1. Bacterial Strains, Plasmids, and Culture Conditions

Herein, the *V. parahaemolyticus* CHN25 strain was employed. *Escherichia coli* DH5α λpir [BEINUO Biotech, Shanghai, China] was used as a host strain for DNA cloning. Conjugation experiments involved the *E. coli β*2155 λpir and pDS132 plasmid, which served as a donor strain and a suicide vector, respectively [24]. For constructing the reverse mutant, the pMMB207 plasmid (Biovector Science Lab, Beijing, China) was utilized as an expression vector [24].

### 4.2. Construction of the Gene Deletion Mutants and Reverse Complementation

Genomic DNA extraction was carried out using the TaKaRa-MiniBEST Bacterial Genomic DNA Extraction Kit (Japan TaKaRa BIO, Dalian Company, Dalian, China). Plasmid DNA was extracted utilizing the TIANpure Midi Plasmid Kit (Tiangen Biotech Beijing Co. Ltd., Beijing, China). Construction of prophage gene deletion and complementary mutants for *V. parahaemolyticus* CHN25 followed a previous method outlined in our earlier studies [24,25]. DNA sequencing was conducted by Sangon in China.

### 4.3. Growth Curve Assay

*V. parahaemolyticus* strains were cultivated in TSB at varying temperatures (15, 25, 37 °C) for 24 h to 60 h intervals. These growth experiments were conducted using the Bioscreen C Automated Growth Curve Analyzer (Lab Systems, Helsinki, Finland). Additionally, growth curves of *V. parahaemolyticus* strains were analyzed in TSB across a spectrum of pH conditions, from pH 5.5 to 8.0 [22,24,25].

### 4.4. Swimming Motility Assays

As previously mentioned, the swimming motility of *V. parahaemolyticus* strains was determined [78,79]. In brief, *V. parahaemolyticus* strains were cultured in TSB at 37 °C to the mid-LGP. A 0.5 μL bacteria solution was pipetted into 0.25% semi-solid TSB. The diameter size was measured and recorded by incubating at 15, 25, and 37 °C for 48, 24, and 12 h, respectively.

### 4.5. Biofilm Formation Assay

As described previously, biofilm formation was determined by crystalline violet staining [80]. Briefly, *V. parahaemolyticus* cultivated in TSB medium at 37 °C was diluted to an OD_600_ of 0.4, and 1 mL of dilution was then inoculated individually into sterile 24-well plates. Planktonic bacteria were removed after incubation at 37 °C for 12, 24, 36, 48, and 60 h. Plates were rinsed 3 times with 1 mL 0.1 M PBS (phosphate-buffered saline, pH 7.2–7.4, Sangon, Shanghai, China). The biofilm was subsequently fixed with 0.1% (*w*/*v*) crystalline violet (Sangon, Shanghai, China). The staining solution was removed and rinsed 3 times with 1 mL PBS each time, dried for 30 min, and then eluted with 1 mL of 95% ethanol for 15 min. A total of 200 μL of the eluate was aspirated in a 96-well plate. The absorbance values were measured at 600 nm using a BioTek Synergy 2 (BioTek, Winooski, VT, USA).

### 4.6. Bacterial Cell Membrane Damage, Hydrophobicity, and Fluidity Assays

Intracellular membrane permeability was determined following a previous method [25]. In brief, 200 μL of bacterial suspension and 2.5 μL 10 mM ONPG solution were added to a 96-well cell culture plate and incubated at 37 °C. The OD_415_ absorbance was measured every 30 min using a BioTek Synergy 2 and labeled as OD_1_; the non-treated suspension was employed as a negative control labeled OD_2_. Determination of cell membrane hydrophobicity and fluidity was carried out according to a previously described method [81].

### 4.7. Human Intestinal Epithelial Cell Viability and Apoptosis Assay

Cell viability of Caco-2 cells infected with V. parahaemolyticus was detected following a previous method [15]. Briefly, Caco-2 cells cultured in DMEM (Dulbecco’s modified eagle medium, Gibco, CA, USA) were inoculated into cell culture plates at 5 × 10^4^ cells/mL/well and incubated at 37 °C and 5% CO_2_ for 24 h. Subsequently, Caco-2 cells were rinsed with 0.1 M PBS (pH 7.2–7.4). At the same time, V. parahaemolyticus cultivated to mid-LGP at 37 °C was collected, washed, and then adjusted to an OD_490_ of 0.2 ± 0.02 with DMEM medium without phenol red. Cell culture plates containing Caco-2 cells were added with 100 µL of bacterial suspension and 10 µL CCK-8 and then incubated with 5% CO_2_ for 4 h at 37 °C. Caco-2 cell viability was determined following a previous method [25]. V. parahaemolyticus-infected Caco-2 cell apoptosis was detected following the method described by Yang and co-workers [25].

### 4.8. Illumina RNA Sequencing

*V. parahaemolyticus* strains were separately cultivated in TSB medium at (3% NaCl, pH 8.5) to mid-LGP. For each Illumina RNA-sequencing experiment, 3 independent RNA specimens were employed. The sequencing was performed using the Illumina HiSeq 2500 platform (Illumina, CA, USA) [15].

### 4.9. Scanning Electron Microscopy (SEM) Analysis

The thermal field emission SEM (Hitachi, 5.0 kV, ×5000; SU5000, Tokyo, Japan) was used to observe and record the cell structure of *V. parahaemolyticus* strains cultivated in TSB to mid-LGP at 37 °C.

### 4.10. Real-Time Reverse Transcription-PCR Assay

RT-qPCR assays were conducted following the methods outlined in a prior work [15]. The 16S rRNA gene was utilized as a housekeeping gene in the RT-qPCR analysis. The mRNA levels of target genes were detected using the 2^−ΔΔCt^ approach. This approach provides a reliable means of quantifying and comparing gene expression levels in the experimental samples (Table S1).

### 4.11. Construction of Recombinant Vectors for Cell Localization Experiments

The *VpaChn25_RS25055* gene was fused to sfGFP to study the localization of *VpaChn25_RS25055* in *V. parahaemolyticus* CHN25 cells [82,83]. sfGFP was synthesized by Sangon and then constructed into the PUC57 vector. The *VpaChn25_RS25055* gene was amplified from the genomic DNA of *V. parahaemolyticus* CHN25 using PCR, employing the RS-sfGFP-F and RS-R primers (Table 1). Simultaneously, the sfGFP gene was amplified from the PUC57 vector, utilizing the sfGFP-F and RS-sfGFP-R primers. These gene fragments, *VpaChn25_RS25055* and sfGFP, were then fused via fusion PCR, creating the *VpaChn25_RS25055*-sfGFP composite fragment. Subsequently, the *VpaChn25_RS25055*-sfGFP was integrated into the *EcoRI* and *XbaI* sites in the expression vector pMMB207 using the infusion technique [84]. This ligated DNA construct was then introduced into *E. coli* DH5α and positive transformants were identified. The recombinant plasmid pMMB207+*VpaChn25_RS25055*-sfGFP was prepared and introduced into the Δ*VpaChn25_RS25055* mutant through electrotransformation. Positive electrotransformants, designated as Δ*VpaChn25_RS25055* (pMMB207+*VpaChn25_RS25055*-sfGFP), were identified via colony PCR utilizing the pMMB207-F/R and *tlh*-F/R primer pairs provided in Table 1. The confirmation process involved the methodologies discussed earlier.

We constructed Δ*VpaChn25_RS25055* (pMMB207+sfGFP) and Δ*VpaChn25_RS25055* (pMMB207) as controls. For Δ*VpaChn25_RS25055* (pMMB207+sfGFP), the sfGFP gene was amplified from the PUC57 vector, utilizing the sfGFP-F2 and sfGFP-R2 primers. Subsequently, the sfGFP gene was integrated into the *EcoRI*/*XbaI* sites in the expression vector pMMB207 using the infusion technique. The ligated DNA was introduced into *E. coli* DH5α, and subsequently, positive transformants were identified through screening. Following this, the modified plasmid pMMB207+sfGFP was prepared and introduced into the Δ*VpaChn25_RS25055* mutant using the electrotransformation procedure detailed earlier. The positively transformed cells, designated as Δ*VpaChn25_RS25055* (pMMB207+sfGFP), were then subjected to screening via colony PCR, employing the pMMB207-F/R and *tlh*-F/R primer pairs outlined in Table 1. Meanwhile, the plasmid PMMB207 was electrotransformed into the Δ*VpaChn25_RS25055* mutant, and the positively transformed cells were designated as Δ*VpaChn25_RS25055* (pMMB207). The confirmation process involved the same methods discussed previously.

### 4.12. Preparation of Cells for Microscopy

A High-Resolution Laser Confocal Microscope (Leica STELLARIS, Wetzlar, Germany) was used to observe and record the cell structure of *V. parahaemolyticus* strains. Briefly, the strains were cultured in TSB at 37 °C to mid-LGP. Then, the cellular morphology was observed using a confocal microscope.

### 4.13. Data Analysis

Sequence analysis was conducted using BLAST (http://www.ncbi.nlm.nih.gov/BLAST (accessed on 5 October 2023)) [24]. Expression of each gene was calculated using RNA-Seq by Expectation-Maximization (RSEM, http://deweylab.github.io/RSEM/ (accessed on 16 May 2023)). Genes with the criteria of fold changes ≥ 2.0 or ≤ 0.5, and *p*-values by BH (fdr correction with Benjamini/Hochberg) < 0.05 relative to the control were defined as DEGs [25]. Gene set enrichment analysis (GSEA) of DEGs was performed against the KEGG database (http://www.genome.jp/kegg/ (accessed on 13 April 2023)). Data were analyzed with SPSS v17.0 (SPSS Inc., Chicago, IL, USA) [25].

## 5. Conclusions

In our study, the single genes *VpaChn25_RS25055*, *VpaChn25_0713*, and *VpaChn25_0714*, which encoded hypothetical proteins in the *V. parahaemolyticus* CHN25 genome, and the continuous three genes *VpaChn25_RS25055-0713-0714* were systematically studied for the first time. We successfully constructed their deletion mutants and complementary mutants. Our data indicated that the deletion of the *VpaChn25_RS25055*, *VpaChn25_0713*, *VpaChn25_0714*, and *VpaChn25_RS25055-0713-0714* genes resulted in a defect in the growth of *V. parahaemolyticus* CHN25 at 15 °C. In addition, the Δ*VpaChn25_RS25055-0713-0714* mutant, deleted with three genes, had a more extended lag phase at pH 5.5–8.0 than the WT and other mutants. The Δ*VpaChn25_0713*, Δ*VpaChn25_RS25055*, and Δ*VpaChn25_RS25055-0713-0714* mutants were also significantly defective in swimming motility at 37, 25, and 15 °C. In our study, the biofilm formation of all four mutants was significantly inhibited, while the Δ*VpaChn25_RS25055* mutant showed significantly less maximum biofilm formation than the other strains (*p* < 0.001). A significant increase in cell membrane fluidity occurred in the three single-gene deletion mutants compared to WT (*p* < 0.01). Meanwhile, the Δ*VpaChn25_RS25055* and Δ*VpaChn25_RS25055-0713-0714* mutants underwent a significant decrease in hydrophobicity. Additionally, it significantly changed only the intracellular membrane permeability of the Δ*VpaChn25_0713* mutant. In the Caco-2 cell model in vitro, the above four deletion mutants showed that the gene deletion significantly reduced the cytotoxicity of *V. parahaemolyticus* CHN25 on human intestinal epithelial cells (*p* < 0.01). The effects of *VpaChn25_RS25055* and *VpaChn25_RS25055-0713-0714* were more significant. We detected 119 *V. parahaemolyticus* strains isolated from aquatic products in Shanghai, China by PCR and found that the homolog genes of *VpaChn25_RS25055*, *VpaChn25_0713*, and *VpaChn25_0714* all had a carrier rate of 1.68% (*n* = 1). For the cellular localization of the prophage gene *VpaChn25_RS25055*, we labeled the *VpaChn25_RS25055* gene with sfGFP and found it localized at both poles of the bacteria cell. These findings revealed that the four prophage-encoded genes in our study increased *V. parahaemolyticus* CHN25’s environmental persistence.

## Figures and Tables

**Table 1:** Oligonucleotide primers used in this study.

Primer	Sequence (5′-3′)	Product Size (bp)	Reference
*VpaChn25_0713*-up-F	GCTCTAGATCACCCTTCACGCTAT	454	This study
*VpaChn25_0713*-up-R	CTCGCTCATTTTCGTTACCCATTGATAGCC		
*VpaChn25_0713*-down-F	GGGTAACGAAAATGAGCGAGACAGCGAGGA	322	This study
*VpaChn25_0713*-down-R	CGAGCTCATTCAGACACTCGCACT		
*VpaChn25_0713*-up-ex-F	TTGGTGGCAAGAAAGG	1673	This study
*VpaChn25_0713*-down-ex-R	ACAAAATCGGGTAGGC		
*VpaChn25_0713*-com-F	CGAGCTCATGGGTAACGAACTGCAACGT	234	This study
*VpaChn25_0713*-com-R	GCTCTAGATTAGGCCGCTTCCTCGCT		
*VpaChn25_RS25055*-up-F	GCTCTAGAACATCGTGACGGTTTAT	491	This study
*VpaChn25_RS25055*-up-R	TCACTCATTTCTTGTTAGGCCGCTTCCTCG		
*VpaChn25_RS25055*-down-F	GCCTAACAAGAAATGAGTGAAGTTAAAGGT	482	This study
*VpaChn25_RS25055*-down-R	CGAGCTCTCATAGCGTTTCCTCTT		
*VpaChn25_RS25055*- up-ex-F	GGCGTTTCTTTCACCT	1983	This study
*VpaChn25_RS25055*-down-ex-R	TCAACAACTTTCGGATT		
*VpaChn25_RS25055*-com-F	CGAGCTCATGAGCGAGACAGCGAGG	186	This study
*VpaChn25_RS25055*-com-R	GCTCTAGATCATTTTTCCCATTCCTT		
*VpaChn25_0714*-up-F	GCTCTAGAACAGCCTTTCCAGATT	308	This study
*VpaChn25_0714*-up-R	AGTTTCATAGTTACCTTTAACTTCACTCAT		
*VpaChn25_0714*-down-F	TTAAAGGTAACTATGAAACTAACCCGTTGC	473	This study
*VpaChn25_0714*-down-R	CGAGCTCACCTACAGCCAGCATT		
*VpaChn25_0714*- up-ex-F	GCAACGAGTGGGATTT	1757	This study
*VpaChn25_0714*-down-ex- R	TTGGTGCTCTGCGGTA		
*VpaChn25_0714*-com-F	CGAGCTCATGAGTGAAGTTAAAGGTAAG	480	This study
*VpaChn25_0714*-com-R	GCTCTAGATCATAGCGTTTCCTCTTTAAG		
*VpaChn25_RS25055-0713-0714*-up-F	GCTCTAGATATCAGAGTCACCCTTCA	468	This study
*VpaChn25_RS25055-0713-0714*-up-R	TTTCCTCTTTTTGCAGTTCGTTACCCATGT		
*VpaChn25_RS25055-0713-0714*-down-F	CGAACTGCAAAAAGAGGAAACGCTATGAAA	485	This study
*VpaChn25_RS25055-0713-0714*-down-R	CGAGCTCACCTACAGCCAGCATT		
*VpaChn25_RS25055-0713-0714* up-ex-F	GGCGTTTCTTTCACCT	1780	This study
*VpaChn25_RS25055-0713-0714*-down-ex-R	CAGCGTATCTTGAGGC		
*VpaChn25_RS25055-0713-0714*-com-F	CGAGCTCATGGGTAACGAACTGCAACGTT	867	This study
*VpaChn25_RS25055-0713-0714*-com-R	GCTCTAGATCATA GCGTTTCCTCTTTAAGGTCTAGG		
RS-sfGFP-F	ACACAGGAAACAGAATTCGTGAAGAGTACGAGGACATGATCAATG		This study
RS-R	TTTTTCCCATTCCTTCTCATTGCTCG		
sfGFP-F	CGAGCAATGAGAAGGAATGGGAAAAACGTGGTTCTGGTGGTGAAGC		This study
RS-sfGFP-R	CTGCAGGTCGACTCTAGATTATTTATATAATTCATCCATACCATGAGTAATACCTGC		
sfGFP-F2	ACACAGGAAACAGAATTCTATGAGCAAAGGAGAAGAACTTTTCACTG		This study
sfGFP-R2	CTGCAGGTCGACTCTAGATTATTTATATAATTCATCCATACCATGAG		
pMMB207-F	GAGCTGTTGACAATTAATCATCGGC		This study
pMMB207-R	CTACGGCGTTTCACTTCTGAGTTC		
*tlh-F*	AAAGCGGATTATGCAGAAGCACTG	596	[15]
*tlh-R*	ACTTTCTAGCATTTTCTCTGC		

## Data Availability

Data are contained within the article or Supplementary Materials. The complete lists of DEGs in the nine strains are available in the NCBI SRA database (http://www.ncbi.nlm.nih.gov/sra/ (accessed on 27 February 2023)) under the accession number PRJNA938975.

## Supplementary Materials

The supporting information can be downloaded at: https://www.mdpi.com/article/10.3390/ijms25031393/s1.
