# Supplementary material for: Biological Function of Prophage-Related Gene Cluster ΔVpaChn25_RS25055~ΔVpaChn25_0714 of Vibrio parahaemolyticus CHN25
Source: Int J Mol Sci. 2024 Jan 23;25(3):1393. doi: 10.3390/ijms25031393 (PMC10855970; doi:10.3390/ijms25031393)
Supplement: Supplementary file 1 [file ijms-25-01393-s001.zip › ijms-2799544-supplementary.pdf]

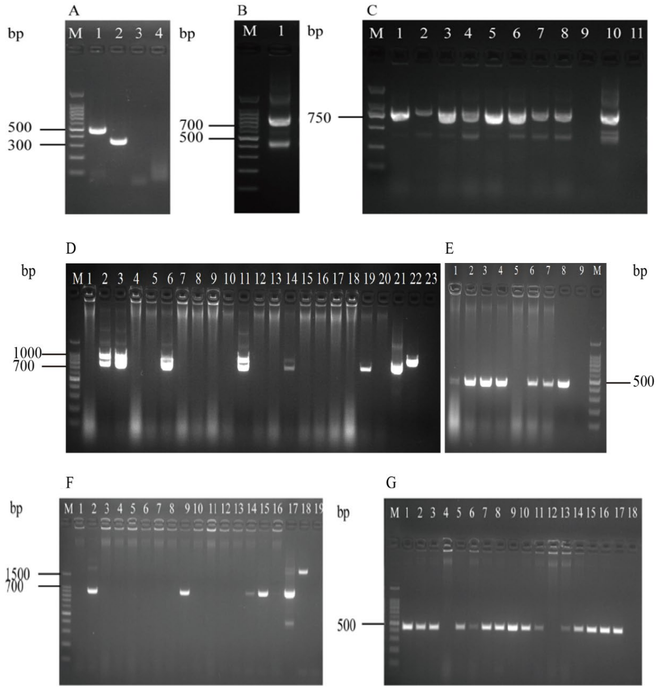

Figure S1. Construction of the Δ*VpaChn25_0713* mutant by agarose gel electrophoresis analysis.

(**A**) Amplicons of upstream and downstream of the *VpaChn25_0713*. M, 100 bp DNA Ladder; Line 1, upstream of *VpaChn25_0713*; Line 2, downstream of *VpaChn25_0713*; Line 3–4, blank. (**B**) Amplicons of upstream& downstream of *VpaChn25_0713*. M, 100 bp DNA Ladder. (**C**) Amplicons of upstream, downstream of *VpaChn25_0713* in the recombinant plasmids. M, D2000 DNA Ladder. (**D**) Amplicons of *VpaChn25_0713*-up-F and *VpaChn25_0713*-down-R. M, 100 bp DNA Ladder. (**E**) Amplicons of *tlh*. M, 100 bp DNA Ladder. (**F**) Amplicons of *VpaChn25_0713*-up-ex-F and *VpaChn25_0713*-down-ex-R. M, 100 bp DNA Ladder. (**G**): Amplicons of *tlh*. M, 100 bp DNA Ladder.

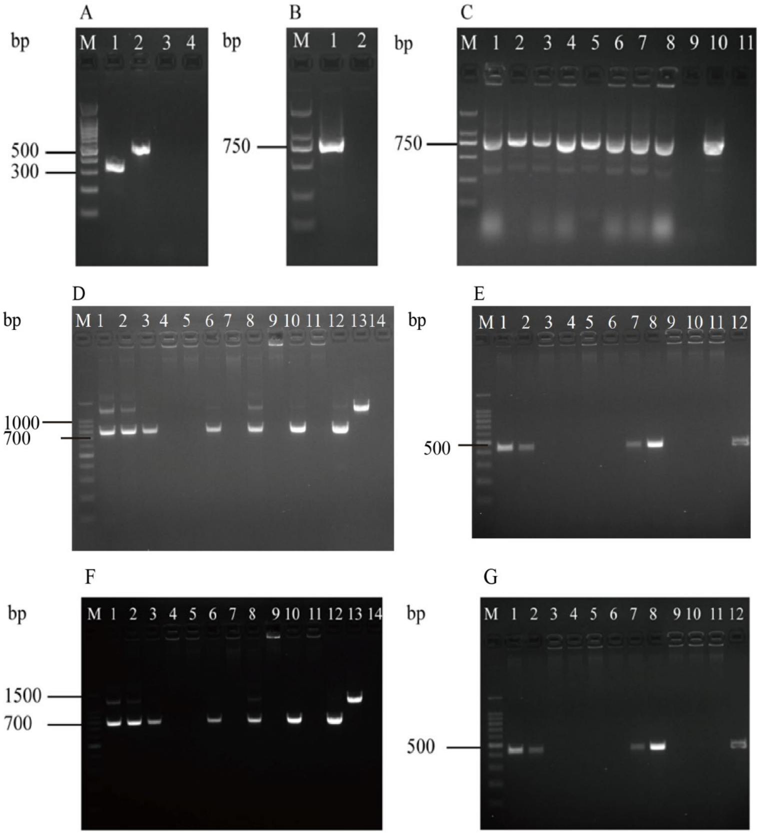

Figure S2. Construction of the Δ*VpaChn25_0714* mutant by agarose gel electrophoresis analysis.

(**A**) amplicons of upstream and downstream of *VpaChn25_0714*. M, 100 bp DNA Ladder; Line 1, upstream of *VpaChn25_0714*; Line 2, downstream of *VpaChn25_0714*; Line 3–4, blank. (**B**) amplicons of upstream& downstream of *VpaChn25_0714*. M, D2000 bp DNA Ladder. (**C**) amplicons of upstream& downstream of *VpaChn25_0714* in the recombinant plasmids. M, D2000 bp DNA Ladder. (**D**) amplicons of *VpaChn25_0714*-up-F and *VpaChn25_0714*-down-R. M, 100 bp DNA Ladder. (**E**): Amplicons of *tlh*. M, 100 bp DNA Ladder. (**F**) Amplicons of *VpaChn25_0714*-up-ex-F and *VpaChn25_0714*-down-ex-R. M, 100 bp DNA Ladder. (**G**) Amplicons of *tlh*. M, 100 bp DNA Ladder.

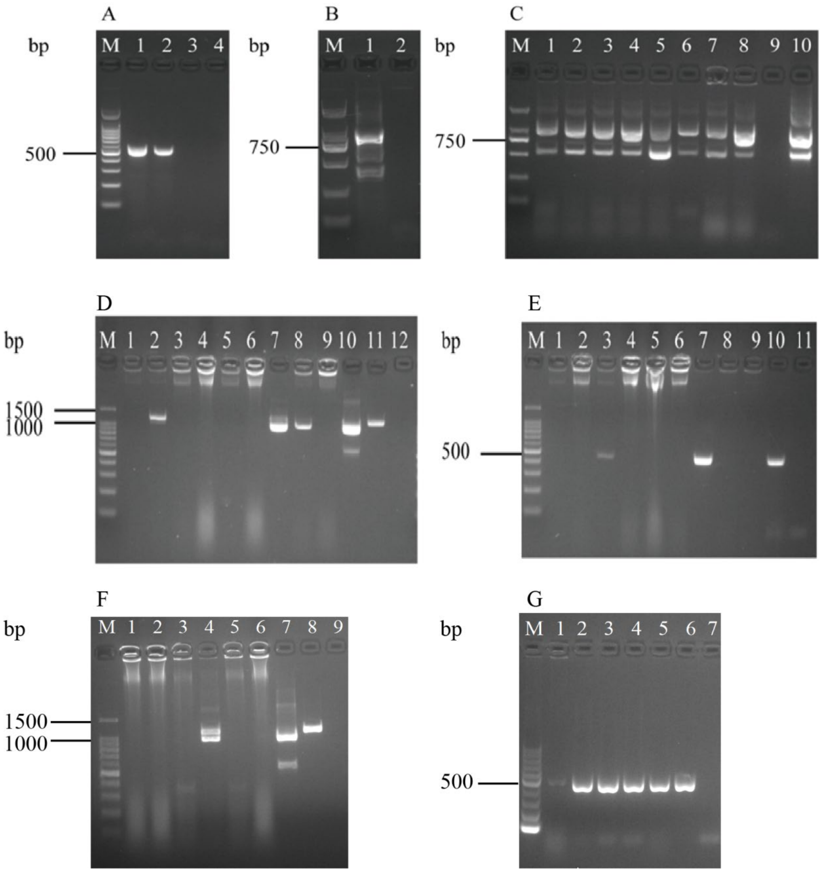

Figure S3. Construction of the Δ*VpaChn25_RS25055* mutant by agarose gel electrophoresis analysis.

(**A**) Amplicons of upstream and downstream of *VpaChn25_RS25055*. M, 100 bp DNA Ladder; Line1, upstream of *VpaChn25_ RS25055*; Line2, downstream of *VpaChn25_ RS25055*; Line3–4, blank. (**B**) Amplicons of upstream& downstream of *VpaChn25_RS25055*. M, D2000 bp DNA Ladder. (**C**) Amplicons of upstream& downstream of *VpaChn25_ RS25055* in the recombinant plasmids. M, D2000 bp DNA Ladder. (**D**) Amplicons of *VpaChn25_ RS25055*-up-F and *VpaChn25_ RS25055*-down-R. M, 100 bp DNA Ladder. (**E**) Amplicons of *tlh*. M, 100 bp DNA Ladder. (**F**) Amplicons of *VpaChn25_ RS25055*-up-ex-F and *VpaChn25_ RS25055*-down-ex-R. M, 100 bp DNA Ladder. (**G**) Amplicons of *tlh*. M, 100 bp DNA Ladder.

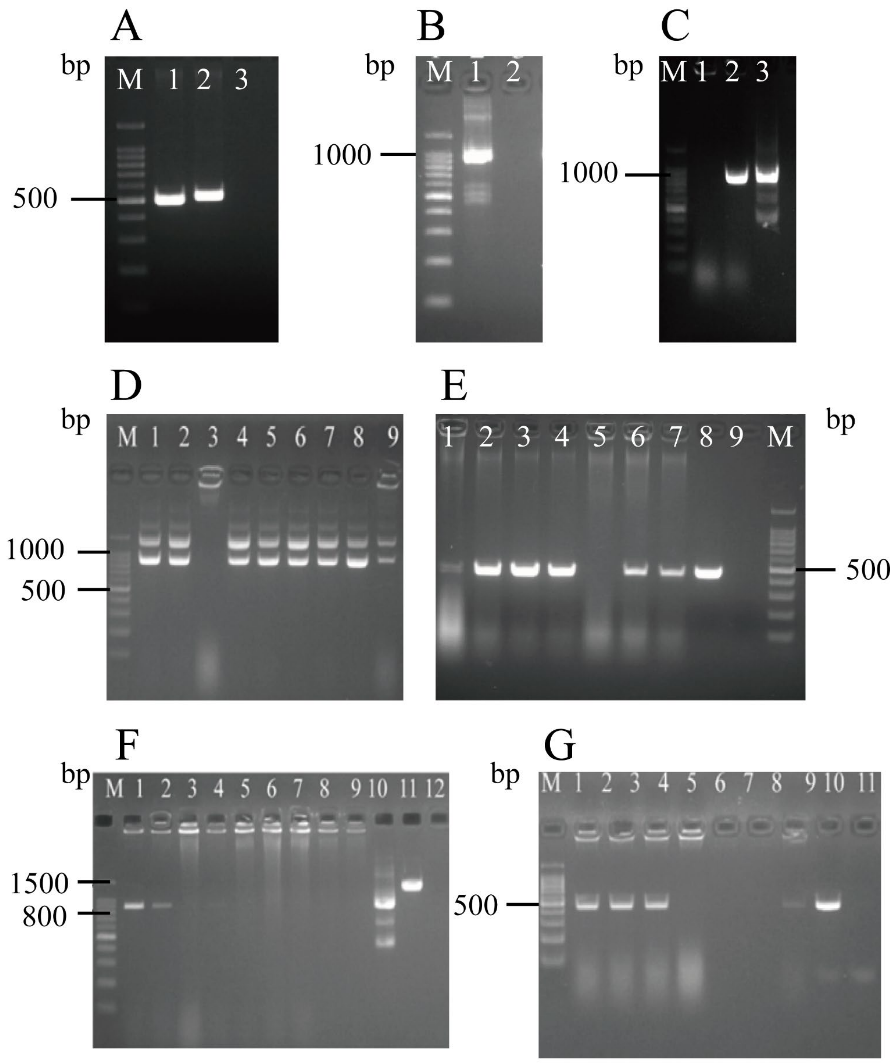

Figure S4. Construction of the Δ*VpaChn25_RS25055-0713-0714* mutant by agarose gel electrophoresis analysis.

(**A**) Amplicons of upstream and downstream of *VpaChn25_RS25055-0713-0714.* M, 100 bp DNA Ladder; Line1, upstream of *VpaChn25_RS25055-0713-08714*; Line2, downstream of *VpaChn25_RS25055-0713-0714*; Line3, blank. (**B**) Amplicons of upstream& downstream of *VpaChn25_RS25055-0713-0714.* M, 100 bp DNA Ladder. (**C**) Amplicons of upstream& downstream of *VpaChn25_RS25055-0713-0714* in the recombinant plasmids. M, 100 bp DNA Ladder. (**D**) Amplicons of *VpaChn25_RS25055-0713-0714*-up-F and *VpaChn25_RS25055-0713-0714*-down-R. M, 100 bp DNA Ladder. (**E**) Amplicons of *tlh*. M, 100 bp DNA Ladder. (**F**) Amplicons of *VpaChn25_RS25055-0713-0714*-up-ex-F and *VpaChn25_RS25055-0713-0714*-down-ex-R. M, 100 bp DNA Ladder. (**G**) Amplicons of *tlh*. M, 100 bp DNA Ladder.

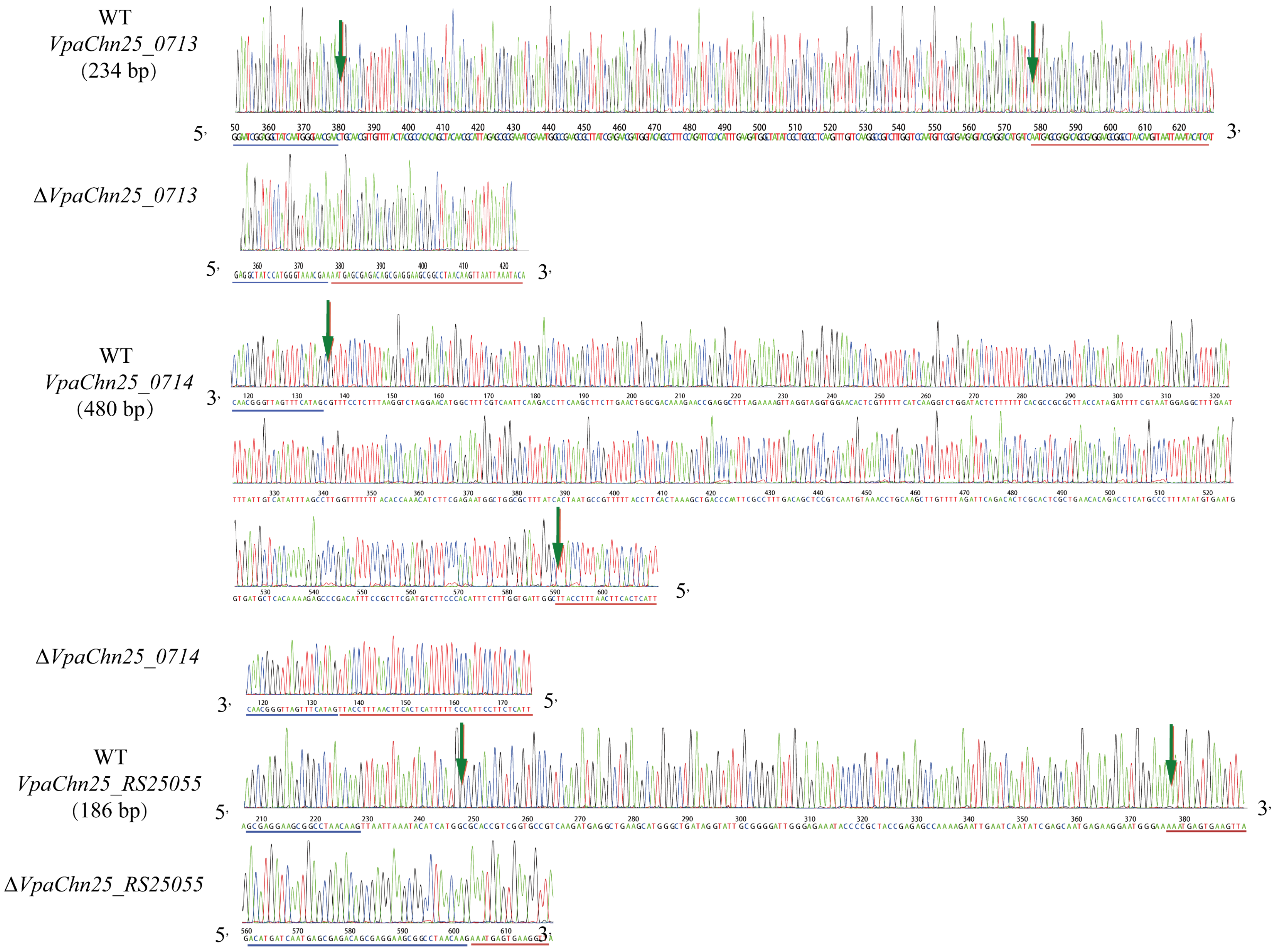
WT
VpaChn25_0713
(234 bp)
ΔVpaChn25_0713
WT
VpaChn25_0714
(480 bp)
ΔVpaChn25_0714
WT
VpaChn25_RS25055
(186 bp)
ΔVpaChn25_RS25055

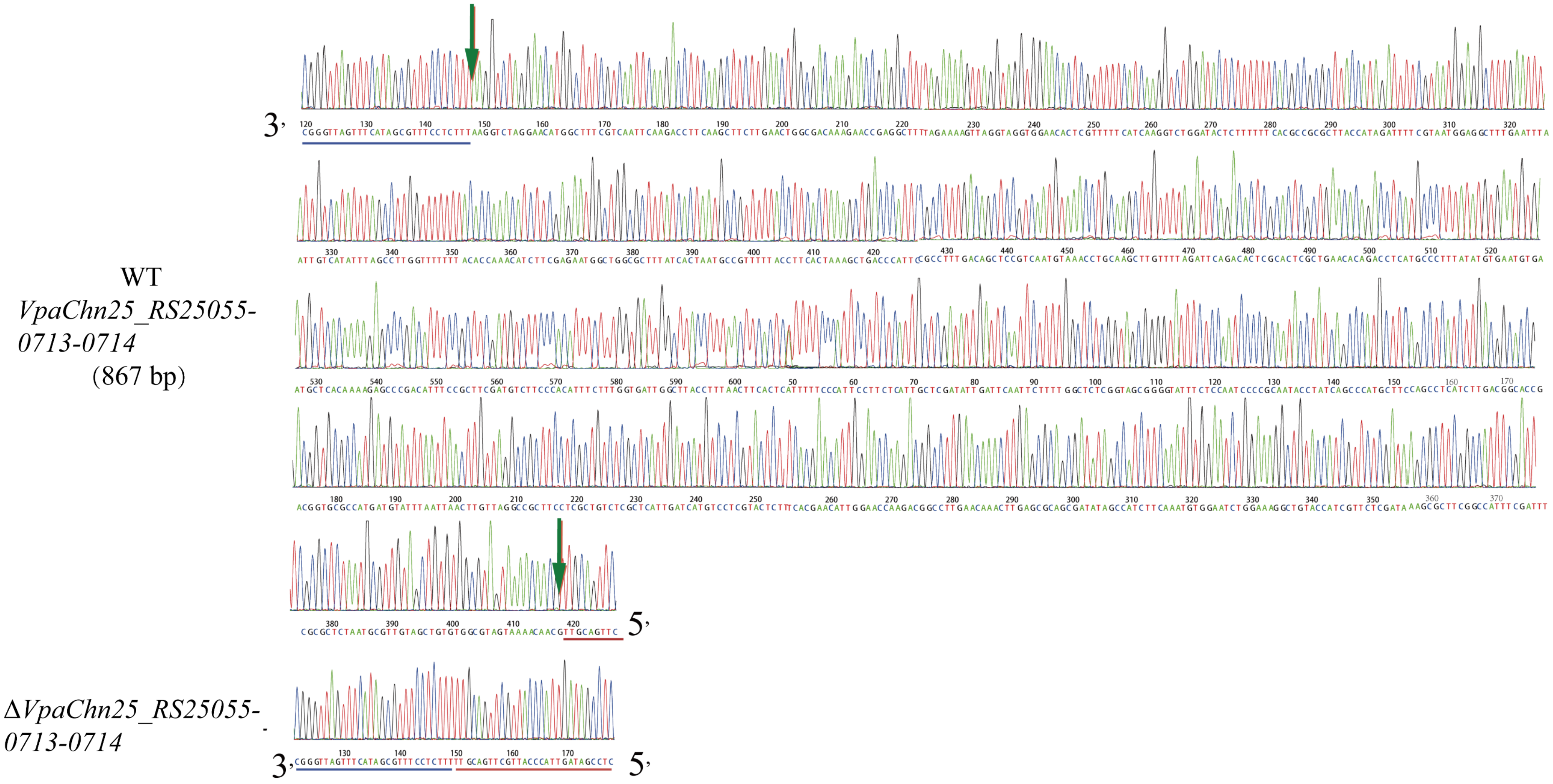

Figure S5. DNA sequencing chromatographs and schematic drawings of the *V. parahaemolyticus* CHN25 WT and Δ*VpaChn25_0713*, Δ*VpaChn25_RS25055*, Δ*VpaChn25_0714*, and Δ*VpaChn25_RS25055-0713-0714* mutants, respectively. The sequence between the two arrows represents the corresponding prophage gene base sequence

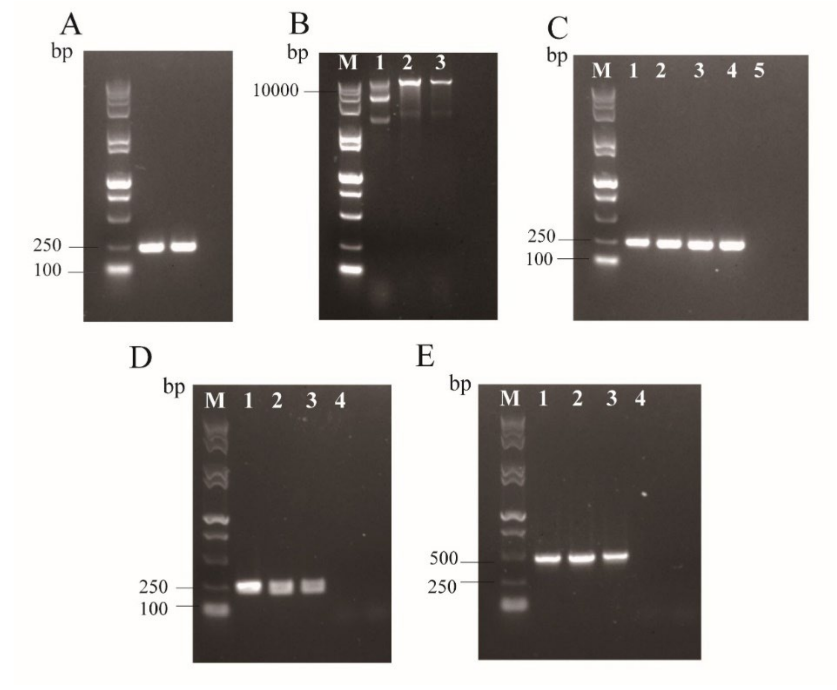

Figure S6. Construction of the Δ*VpaChn25_0713*-com by agarose gel electrophoresis analysis.

(**A**) Amplicons of the *VpaChn25_0713* gene. M, D15000+2000 Ladder; Lines 1–2, positive bands; Line 3, blank control. (**B**) High-level expression vector pMMB207 plasmid DNA and its double digestion results. M, D15000+2000 Ladder. (**C**) Double digestion of the recombinant pMMB207+*VpaChn25_0713* plasmid. M, D15000+2000 Ladder. (**D**) Amplicons of *VpaChn25_0713* from positive transformants. M, D15000+2000 Ladder. (**E**) Amplicons of *tlh* from Δ*VpaChn25_0713*-com mutants. M, D15000+2000 Ladder.

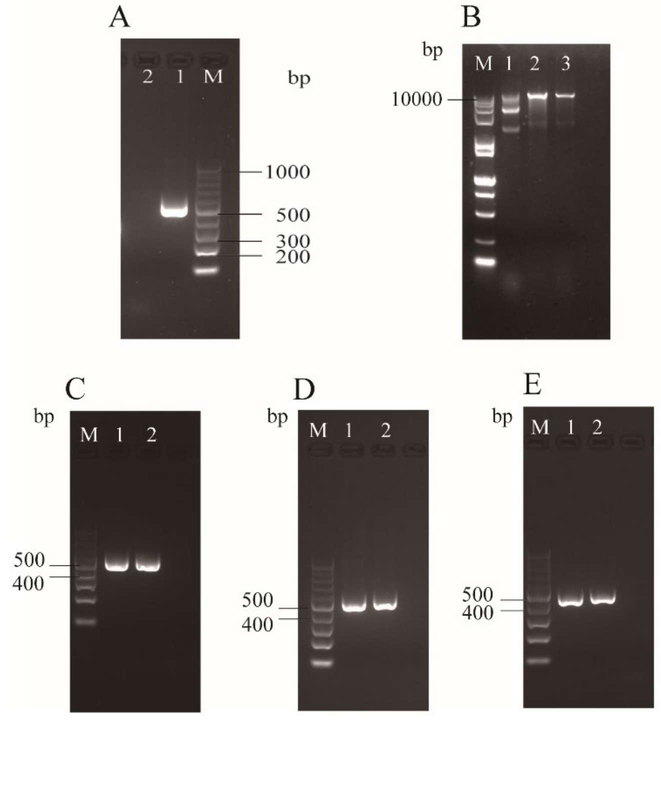

Figure S7. Construction of the Δ*VpaChn25_0714*-com by agarose gel electrophoresis analysis.

(**A**) Amplicons of the *VpaChn25_0714* gene. M, 100 bp DNA Ladder; Lines 1, positive bands; Line 2, blank control. (**B**) High level expression vector pMMB207 plasmid DNA and its double digestion results. M, D15000+2000 Ladder. (**C**) Double digestion of the recombinant pMMB207+*VpaChn25_0714* plasmid. M, 100 bp DNA Ladder. (**D**) Amplicons of *VpaChn25_0714* from positive transformants. M, 100 bp DNA Ladder. (**E**) Amplicons of *tlh* from Δ*VpaChn25_0714*-com mutants. M, 100 bp DNA Ladder.

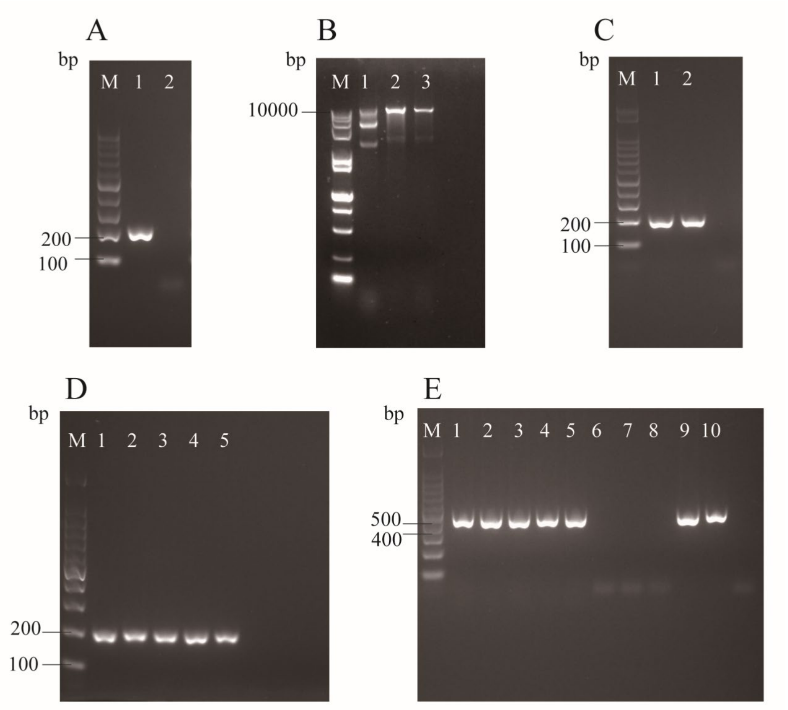

Figure S8. Construction of the Δ*VpaChn25_RS25055*-com by agarose gel electrophoresis analysis.

(**A**) Amplicons of the *VpaChn25_RS25055* gene. M, 100 bp DNA Ladder; Lines 1, positive bands; Line 2, blank control. (**B**) High level expression vector pMMB207 plasmid DNA and its double digestion results. M, D15000+2000 Ladder. (**C**) Double digestion of the recombinant pMMB207+*VpaChn25_RS25055* plasmid. M, 100 bp DNA Ladder. (**D**) Amplicons of *VpaChn25_RS25055* from positive transformants. M, 100 bp DNA Ladder. (**E**) Amplicons of *tlh* from Δ*VpaChn25_RS25055*-com mutants. M, 100 bp DNA Ladder.

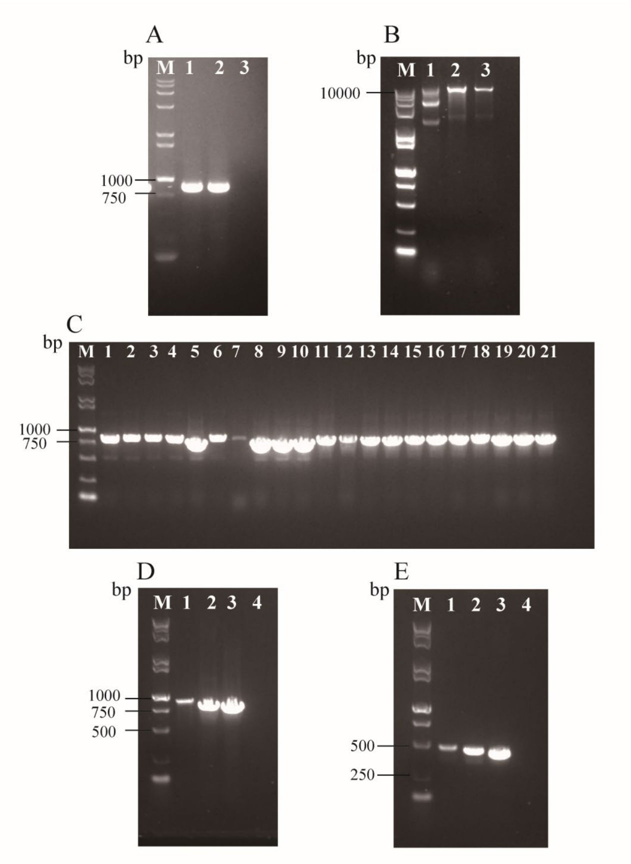

Figure S9. Construction of the Δ*VpaChn25_RS25055-0713-0714*-com by agarose gel electrophoresis analysis.

(**A**) Amplicons of the *VpaChn25_RS25055-0713-0714* gene. M, D15000+2000 Ladder; Lines 1, positive bands; Line 2, blank control. (**B**) High level expression vector pMMB207 plasmid DNA and its double digestion results. M, D15000+2000 Ladder. (**C**) Double digestion of the recombinant pMMB207+*VpaChn25_RS25055-0713-0714* plasmid. M, D15000+2000 Ladder. (**D**) Amplicons of *VpaChn25_RS25055-0713-0714* from positive transformants. M, D15000+2000 Ladder. (**E**) Amplicons of *tlh* from Δ*VpaChn25_RS25055-0713-0714*-com mutants. M, D15000+2000 Ladder

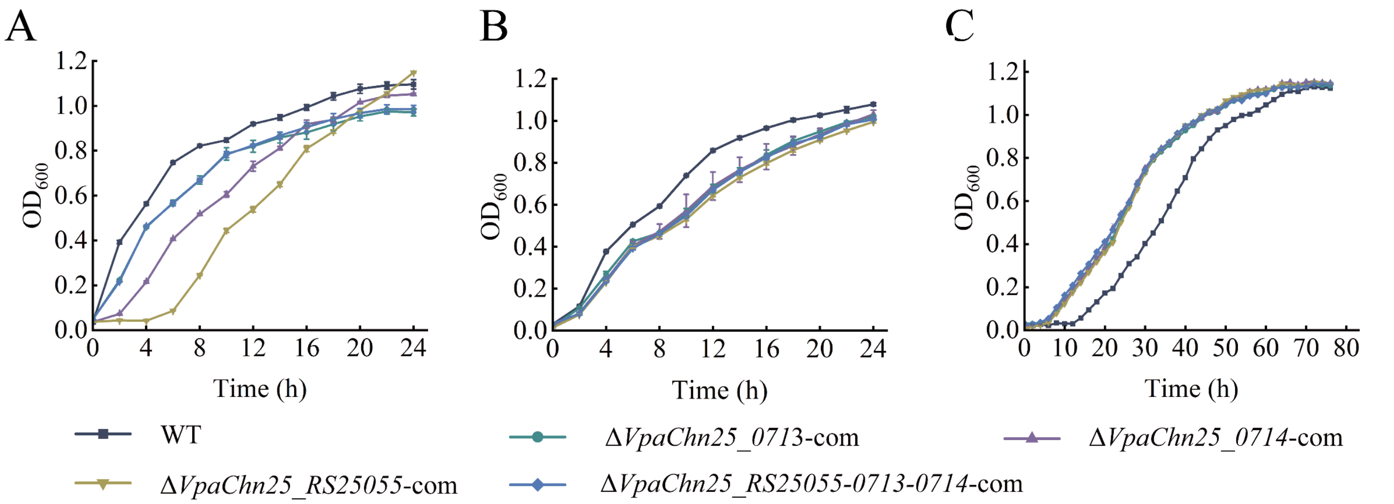

Figure S10. Survival of the *V. parahaemolyticus* CHN25 (WT), Δ*VpaChn25_RS25055*-com, Δ*VpaChn25_0713*-com, Δ*VpaChn25_0714*-com, and Δ*VpaChn25_RS25055-0713-0714*-com strains at different temperatures. The strains were individually incubated in the TSB medium (pH 8.5, 3% NaCl) at 37 °C (**A**), 25 °C (**B**), and 15 °C (**C**), respectively.

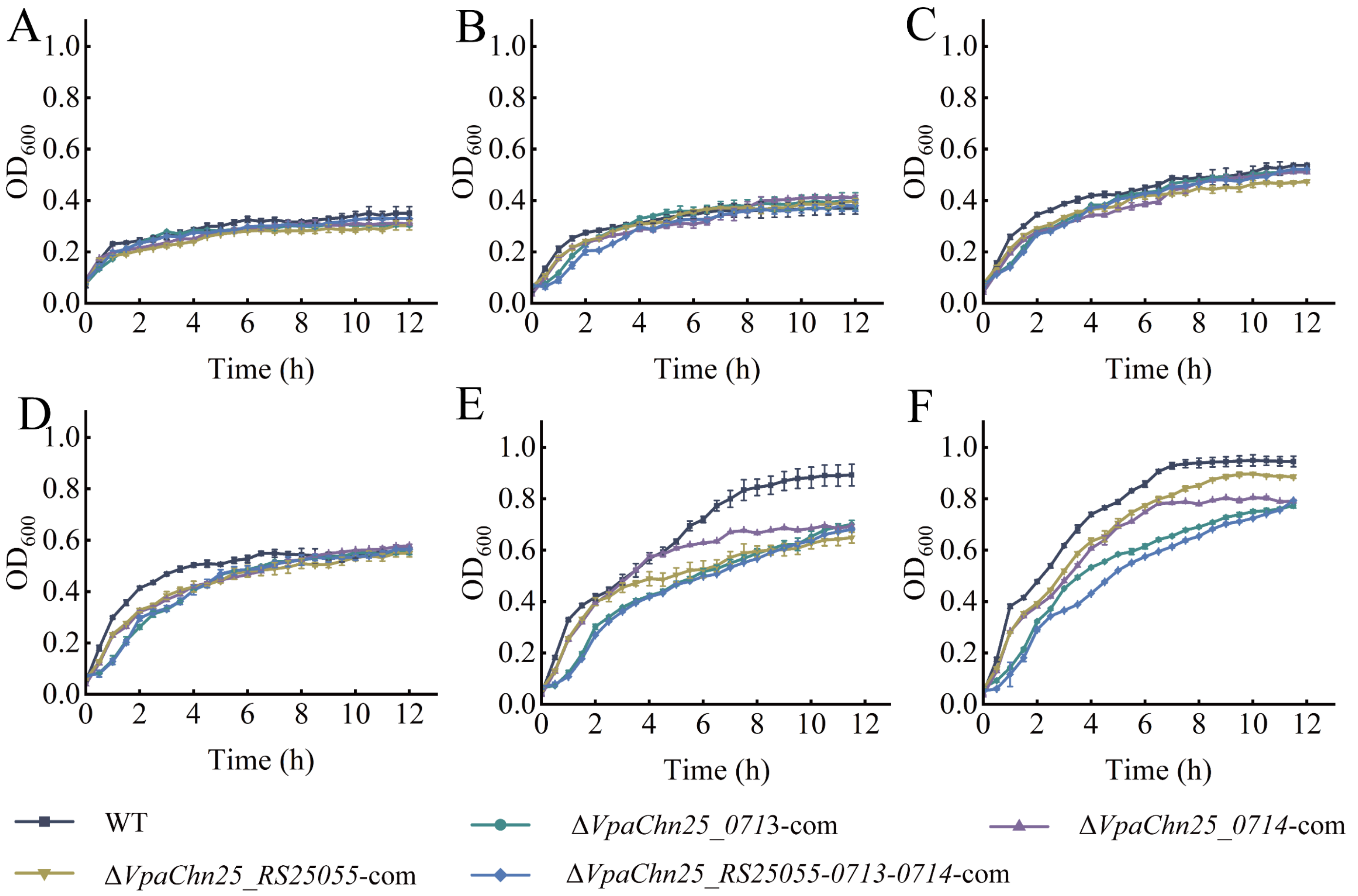

Figure S11. Survival of the *V. parahaemolyticus* CHN25 WT, Δ*VpaChn25_RS25055*-com, Δ*VpaChn25_0713*-com, Δ*VpaChn25_0714*-com, Δ*VpaChn25_RS25055-0713-0714*-com strains at different pH conditions. The strains were individually incubated in the TSB medium (pH 8.5, 3% NaCl) at pH 5.5 (**A**), pH 6.0 (**B**), pH 6.5 (**C**), pH 7.0 (**D**), pH 7.5 (**E**), and pH 8.0 (**F**), respectively.

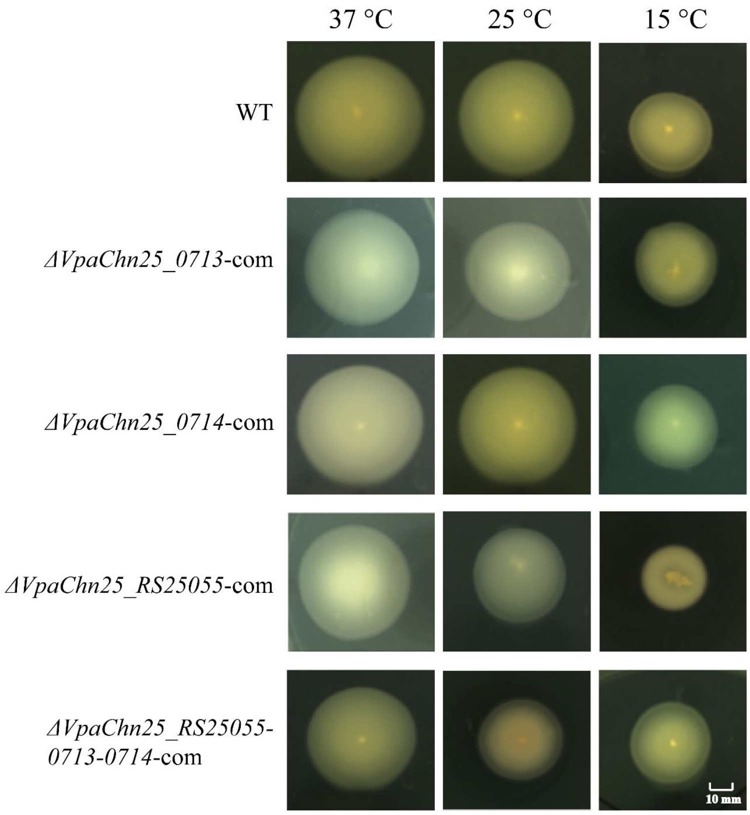

Figure S12 Swimming motility of the *V. parahaemolyticus* CHN25 WT, Δ*VpaChn25_RS25055*-com, Δ*VpaChn25_0713*-com, Δ*VpaChn25_0714*-com, Δ*VpaChn25_RS25055-0713-0714*-com strains at different temperatures. The strains were individually incubated in a semi-solid TSB medium containing 0.25% agar at 37 °C, 25 °C, and 15 °C, respectively.

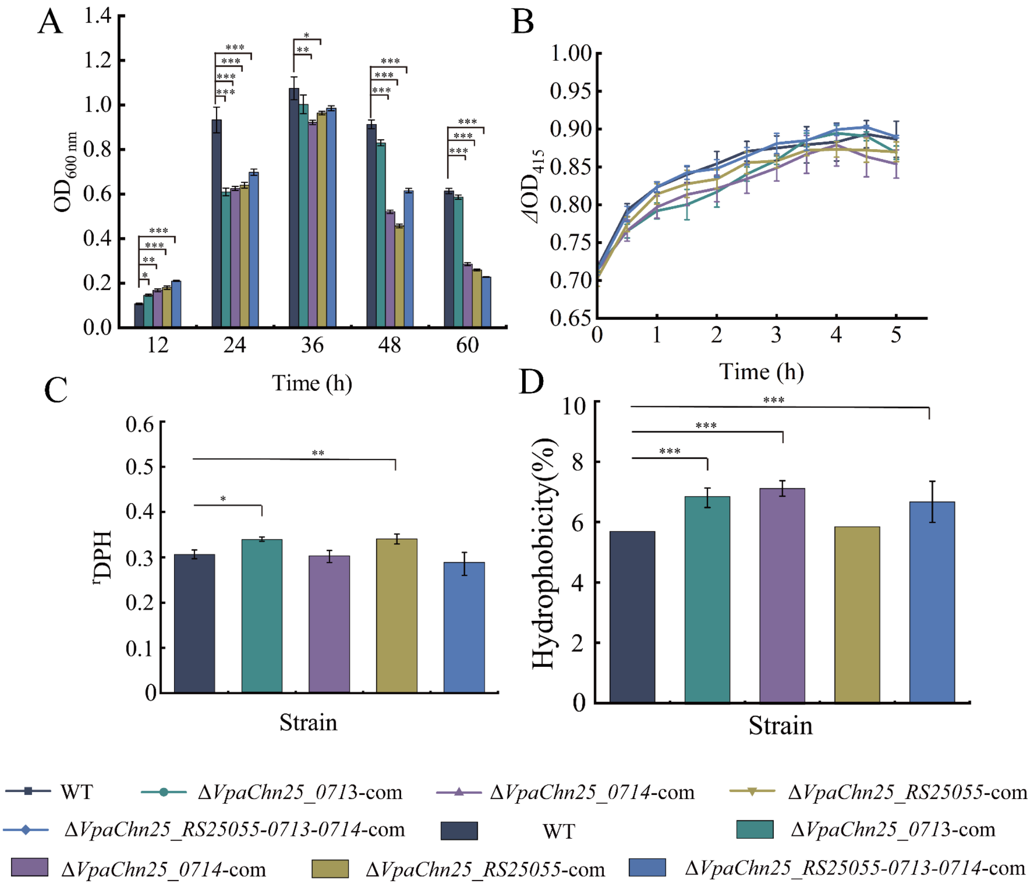

Figure S13. Biofilm formation (**A**), internal membrane permeability (**B**), fluidity (**C**), and hydrophobicity (**D**) of the *V. parahaemolyticus* CHN25 (WT), Δ*VpaChn25_RS25055*-com, Δ*VpaChn25_0713*-com, Δ*VpaChn25_0714*-com, Δ*VpaChn25_RS25055-0713-0714*-com strains. DPH: 1, 6-diphenyl-1,3,5-hexatriene. *$p < 0.05$, **$p < 0.01$, ***$p < 0.001$.

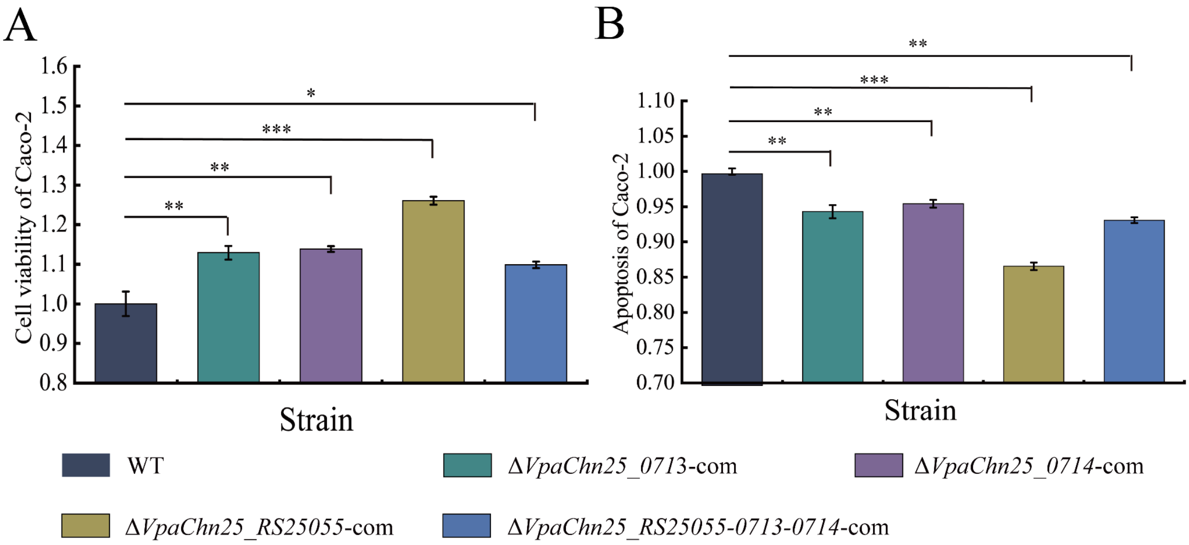

Figure S14. The viability and apoptosis of Caco-2 cells infected by the *V. parahaemolyticus* CHN25 WT, Δ*VpaChn25_RS25055*-com, Δ*VpaChn25_0713*-com, Δ*VpaChn25_0714*-com, and Δ*VpaChn25_RS25055-0713-0714*-com strains. The infection was performed at 37 °C for 4 h. (**A**) Cell viability; (**B**) cell apoptosis. *$p < 0.05$, **$p < 0.01$, ***$p < 0.001$.

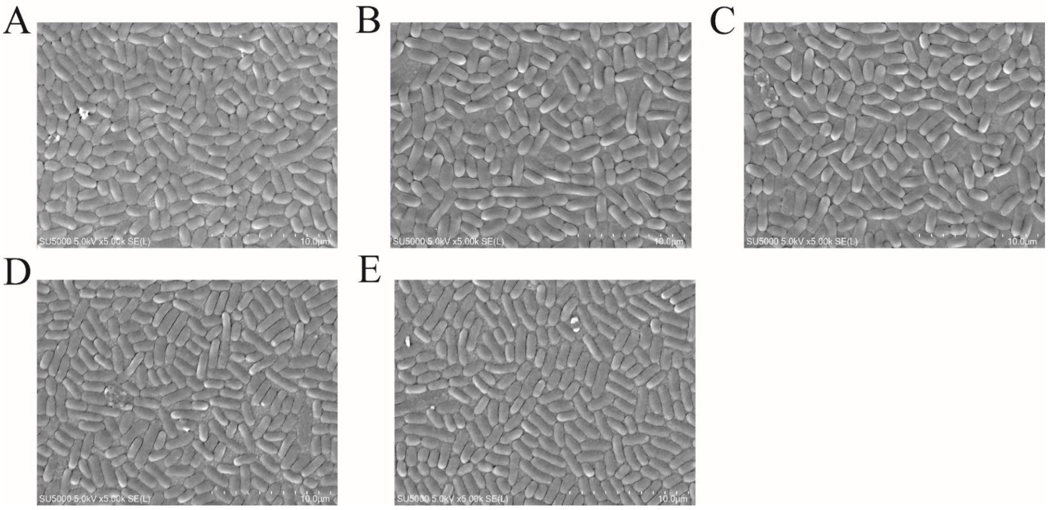

Figure S15. The SEM observation of cell structure of the *V. parahaemolyticus* CHN25 (WT), Δ*VpaChn25_RS25055*-com, Δ*VpaChn25_0713*-com, Δ*VpaChn25_0714*-com, and Δ*VpaChn25_RS25055-0713-0714*-com strains. (**A**) WT; (**B**) Δ*VpaChn25_0713*-com; (**C**) Δ*VpaChn25_0714*-com; (**D**) Δ*VpaChn25_RS25055*-com; (**E**) Δ*VpaChn25_RS25055-0713-0714*-com.

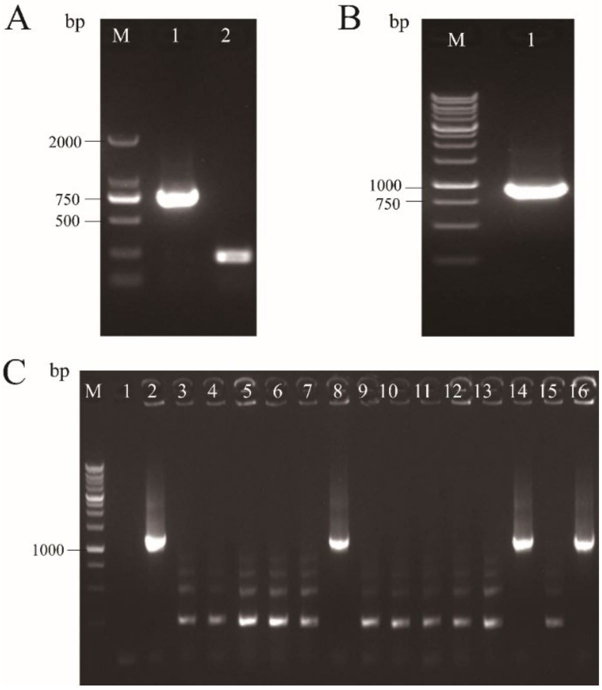

Figure S16. Construction of the Δ*VpaChn25_RS25055* (pMMB207+*VpaChn25_RS25055*-sfGFP) by agarose gel electrophoresis analysis.

(**A**) Amplicons of the *VpaChn25_RS25055* and sfGFP genes. M, 2000 bp DNA Ladder; Lines 1, sfGFP; Line 2, *VpaChn25_RS25055*. (**B**) PCR fusion product of *VpaChn25_RS25055* and sfGFP genes. M, DL 1000 bp DNA Ladder. (**C**) Ligation of the high expression vector pMMB207 and *VpaChn25_RS25055*+sfGFP. M, DL 1000 bp DNA Ladder; Line 1–15, amplification results of clones 1–15.

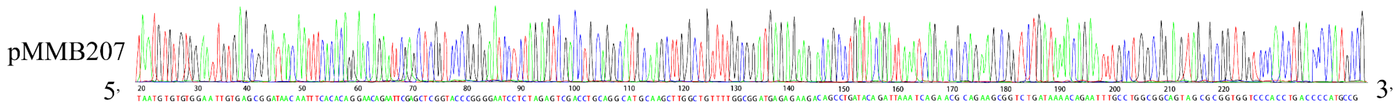

Figure S17. DNA sequencing chromatographs and schematic drawings of Δ*VpaChn25_RS25055* (pMMB207)

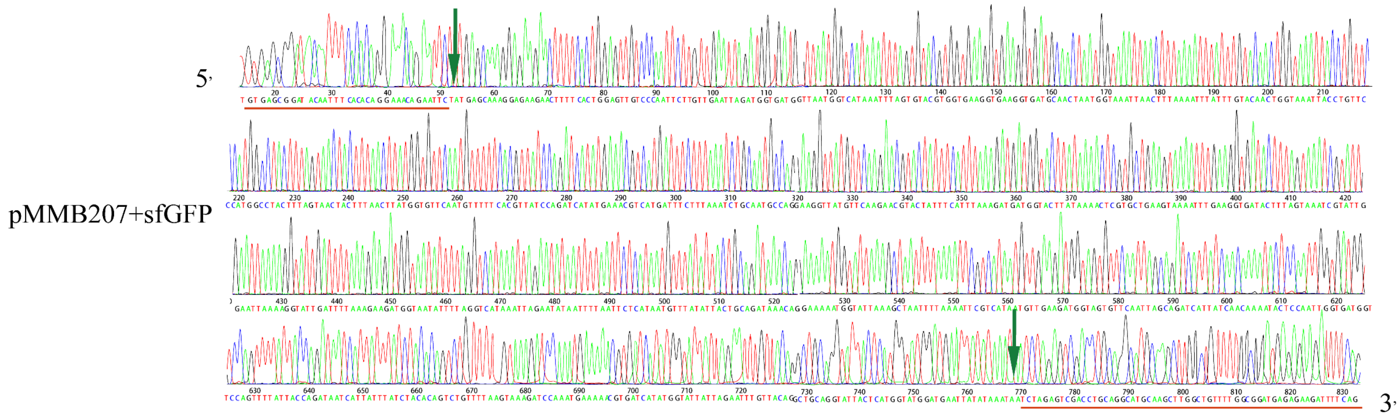

Figure S18. DNA sequencing chromatographs and schematic drawings of Δ*VpaChn25_RS25055* (pMMB207+ sfGFP). The sequence between the two green arrows represents sfGFP.

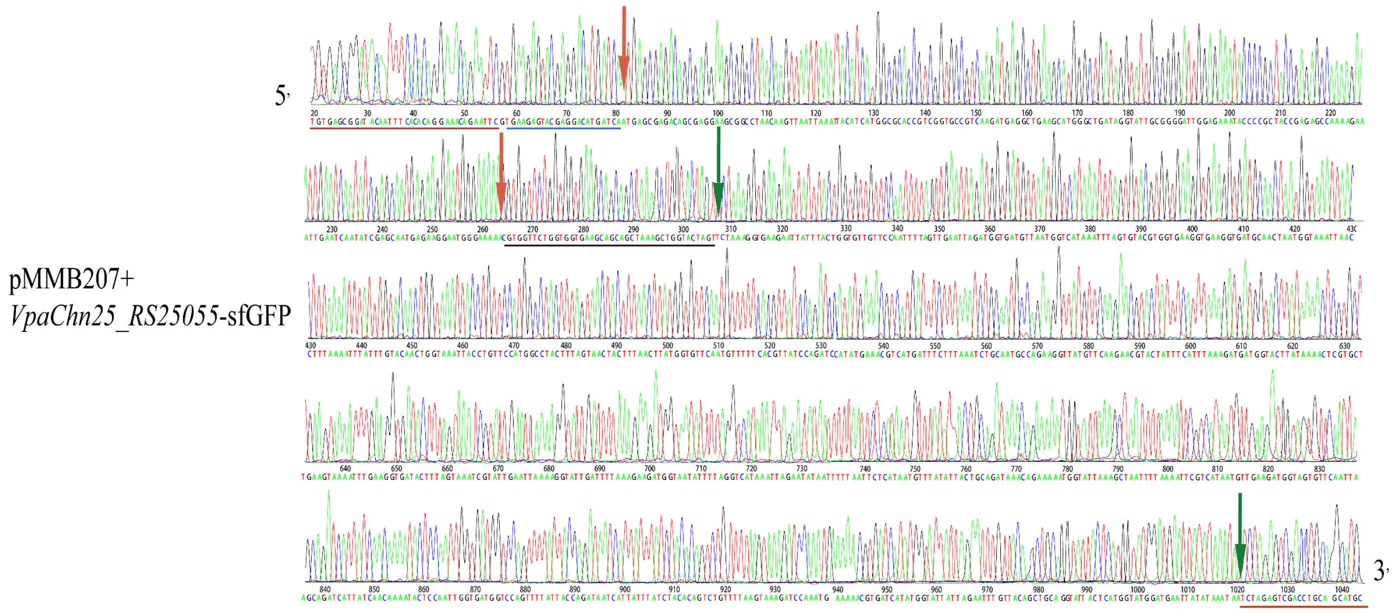

Figure S19. DNA sequencing chromatographs and schematic drawings of Δ*VpaChn25_RS25055* (pMMB207+*VpaChn25_RS25055*-sfGFP). The sequence between two red arrows represents *VpaChn25_RS25055* and the sequence between two green arrows represents sfGFP.

Table S1. Oligonucleotide primers used in RT-PCR assay

	Primer	Sequence(5′-3′)	Product size (bp)
Δ*VpaChn25_0713*	*VpaChn25_RS05195*-F	GCACTAAACGCAATCCACT	283
	VpaChn25_RS05195-R	TGCCCATAAATACAGAAACA	
	VpaChn25_RS11110-F	AACCTAGAAAGGCCGAATG	229
	VpaChn25_RS11110-R	CACGAAATCCAAGACTGCT	
	VpaChn25_RS23525-F	CCAGCAGACTTCACATTCG	196
	VpaChn25_RS23525-R	TGATGTTGCCTGTTTGGTC	
	VpaChn25_RS22165-F	ACAGTGGCTCACCCAGACG	232
	VpaChn25_RS22165-R	GGGACTCAACCGCAACAGG	
	VpaChn25_RS21450-F	AATCAAGCGAAATCAAAGAG	201
	VpaChn25_RS21450-R	AAGCGTTAAGCATCCACAT	
	VpaChn25_RS23335-F	GTGGTATGGACGGAAGCAA	243
	VpaChn25_RS23335-R	GAACCCAAGCGAAAGAAAC	
Δ*VpaChn25_0714*	*VpaChn25_RS10475*-F	TACTTCCGTCGTGGTAGCC	210
	VpaChn25_RS10475-R	CATTTGAGCCGCACCTTTT	
	VpaChn25_RS15965-F	CGCCAACGGAGTTCGGTAA	182
	VpaChn25_RS15965-R	CGCCAGCATCGCAATCTTT	
	VpaChn25_RS05195-F	GCACTAAACGCAATCCACT	283
	VpaChn25_RS05195-R	TGCCCATAAATACAGAAACA	
	VpaChn25_RS11110-F	AACCTAGAAAGGCCGAATG	229
	VpaChn25_RS11110-R	CACGAAATCCAAGACTGCT	
	VpaChn25_RS10585-F	CGTATCGGTCGTTTCGTAT	226
	VpaChn25_RS10585-R	CGTCCCATTTAAGGTCAGC	
	VpaChn25_RS00065-F	CCCAAAGAGTCAGAGTTATT	122
	VpaChn25_RS00065-R	CAACCGTCAAGCCAGTAAG	
Δ*VpaChn25_RS25055*	*VpaChn25_RS02420*-F	AACGACGAACTACTAAACTC	256
	VpaChn25_RS02420-R	TAACCAGAAACACGGATTG	
	VpaChn25_RS00565-F	CTTCTATCCGTATCCCAGTG	119
	VpaChn25_RS00565-R	GTCAAGACCAGCCATCAGT	
	VpaChn25_RS18370-F	TCAACATGATGGGAACTGG	106
	VpaChn25_RS18370-R	TAGCGTCTGCTGATAGTAGGA	
	VpaChn25_RS11110-F	AACCTAGAAAGGCCGAATG	229
	VpaChn25_RS11110-R	CACGAAATCCAAGACTGCT	
	VpaChn25_RS05045-F	GCCTACACTTCGCTCCAAT	178
	VpaChn25_RS05045-R	CGCTTCACAACACGACCAG	
	VpaChn25_RS11070-F	AGCGTTTGTCTTCGGGTTA	295
	VpaChn25_RS11070-R	TCAGCGATACGGTTTAGTT	
Δ*VpaChn25_RS25055-0713-0714*	*VpaChn25_RS01880*-F	GAATCGCAAACGGATACAG	207
	VpaChn25_RS01880-R	CACCAAAACCGATCACGATTGGGCT	
	VpaChn25_RS01875-F	CATCCCTACGGAAGTTATT	125
	VpaChn25_RS01875-R	TGCTACCGCTGTCTATCAC	
	VpaChn25_RS05195-F	GCACTAAACGCAATCCACT	283
	VpaChn25_RS05195-R	TGCCCATAAATACAGAAACA	

VpaChn25_RS11110-F	AACCTAGAAAGGCCGAATG	229
VpaChn25_RS11110-R	CACGAAATCCAAGACTGCT	
VpaChn25_RS16665-F	GTTACTGTAGATGCGAAAGG	121
VpaChn25_RS16665-R	CAACAACAAGACGACCAAG	
VpaChn25_RS22165-F	ACAGTGGCTCACCCAGACG	232
VpaChn25_RS22165-R	GGGACTCAACCGCAACAGG	

Table S2. Expression of representative DEGs in Δ*VpaChn25_0713*, Δ*VpaChn25_0714*, Δ*VpaChn25_RS25055*, and Δ*VpaChn25_RS25055-0713-0714* mutants by the RT-PCR assay.

strain	Gene	Predicted protein	Fold change	
			RNA-Seq.	RT-PCR
Δ*VpaChn25_0713*	*VpaChn25_RS05195*	formate C-acetyltransferase	0.162	0.306
	VpaChn25_RS11110	helix-turn-helix domain-containing protein	0.194	0.113
	VpaChn25_RS23525	alkyl hydroperoxide reductase subunit C	0.262	0.475
	VpaChn25_RS22165	maltose/maltodextrin ABC transporter	4.423	2.156
	VpaChn25_RS21450	DNA starvation/stationary phase protection	0.412	0.578
	VpaChn25_RS23335	maltoporin	3.497	2.986
Δ*VpaChn25_0714*	*VpaChn25_RS10475*	bifunctional acetaldehyde-CoA/alcohol dehydrogenase	0.396	0.478
	VpaChn25_RS15965	DUF3081 domain-containing protein	2.462	3.579
	VpaChn25_RS05195	formate C-acetyltransferase	0.172	0.223
	VpaChn25_RS11110	helix-turn-helix domain-containing protein	0.246	0.378
	VpaChn25_RS10585	type I glyceraldehyde-3-phosphate dehydrogenase	0.285	0.872
	VpaChn25_RS00065	hypothetical protein	2.679	1.689
Δ*VpaChn25_RS25055*	*VpaChn25_RS02420*	autonomous glycyl radical cofactor GrcA	0.444	0.372
	VpaChn25_RS00565	glutamate--ammonia ligase	6.026	4.358
	VpaChn25_RS18370	hypothetical protein	2.786	2.323
	VpaChn25_RS11110	helix-turn-helix domain-containing protein	0.188	0.372
	VpaChn25_RS05045	uridine phosphorylase	0.317	0.742
	VpaChn25_RS11070	flagellin	0.396	0.562
Δ*VpaChn25_RS25055-0713-0714*	*VpaChn25_RS01880*	glutamine-fructose-6-phosphate transaminase (isomerizing)	0.237	0.676
	VpaChn25_RS01875	DeoR family transcriptional regulator	0.237	0.242
	VpaChn25_RS05195	formate C-acetyltransferase	0.055	0.469
	VpaChn25_RS11110	helix-turn-helix domain-containing protein	0.129	0.187
	VpaChn25_RS16665	porin	3.477	4.552
	VpaChn25_RS22165	maltose/maltodextrin ABC transporter substrate-binding protein MalE	7.184	8.135

Table S3. Major changed metabolic pathways in the Δ*VpaChn25_0713* mutant.

Metabolism Pathway	Gene ID	Fold Change	Gene Description
Phosphotransferase system	*VpaChn25_RS19520*	0.057	PTS fructose transporter subunit IIBC
	VpaChn25_RS19525	0.080	1-phosphofructokinase
	VpaChn25_RS19530	0.136	Fused PTS fructose transporter subunit IIA/HPr protein
	VpaChn25_RS16970	0.173	PTS sugar transporter subunit IIB
	VpaChn25_RS21735	0.180	PTS sugar transporter subunit IIA

	VpaChn25_RS01935	0.357	PTS mannitol transporter subunit IICBA
	VpaChn25_RS13465	0.370	PTS lactose/cellobiose transporter subunit IIA
	VpaChn25_RS21730	0.451	PTS mannitol transporter subunit IICB
	VpaChn25_RS16965	0.491	PTS ascorbate transporter subunit IIC
	VpaChn25_RS13470	2.022	PTS sugar transporter subunit IIC
	VpaChn25_RS01915	2.370	Phosphoenolpyruvate-protein phosphotransferase
	VpaChn25_RS23450	4.023	Glucose-specific PTS transporter subunit IIBC
Glycolysis/Gluconeogenesis	*VpaChn25_RS10585*	0.203	Type I glyceraldehyde-3-phosphate dehydrogenase
	VpaChn25_RS10590	0.269	D-hexose-6-phosphate mutarotase
	VpaChn25_RS10475	0.306	Bifunctional acetaldehyde-CoA/alcohol dehydrogenase
	VpaChn25_RS19575	0.421	Pyruvate kinase
	VpaChn25_RS23575	0.454	6-phospho-β-glucosidase
	VpaChn25_RS01325	0.492	Triose-phosphate isomerase
	VpaChn25_RS17630	0.494	Phosphoenolpyruvate synthase
	VpaChn25_RS00605	2.174	Phosphoenolpyruvate carboxykinase (ATP)
	VpaChn25_RS18050	2.971	Glyceraldehyde-3-phosphate dehydrogenase
	VpaChn25_RS08880	4.278	Aldehyde dehydrogenase
Glycerolipid metabolism	*VpaChn25_RS16340*	2.077	Glycerate kinase
	VpaChn25_RS01910	2.225	Dihydroxyacetone kinase ADP-binding subunit DhaL
	VpaChn25_RS01905	2.231	Dihydroxyacetone kinase subunit DhaK
	VpaChn25_RS06325	2.240	Triacylglycerol lipase
	VpaChn25_RS01900	3.040	Glycerol dehydrogenase
	VpaChn25_RS17910	3.844	Diacylglycerol kinase
Sulfur metabolism	*VpaChn25_RS07520*	0.150	Dimethylsulfoxide reductase subunit B
	VpaChn25_RS07525	0.172	Dimethyl sulfoxide reductase anchor subunit
	VpaChn25_RS07515	0.211	Dimethyl sulfoxide reductase subunit A
	VpaChn25_RS01595	0.228	Adenylyl-sulfate kinase
	VpaChn25_RS01585	0.358	Sulfate adenylyltransferase subunit CysN
	VpaChn25_RS07355	0.497	Homoserine O-succinyltransferase
	VpaChn25_RS04200	0.498	Cysteine synthase A
	VpaChn25_RS13920	0.499	Phosphoadenylyl-sulfate reductase
Mannose and fructose metabolism	*VpaChn25_RS22285*	0.475	Mannose-6-phosphate isomerase, class I
Oxidative phosphorylation	*VpaChn25_RS05680*	0.266	Cytochrome bd-I oxidase subunit CydX
	VpaChn25_RS20880	0.308	Cytochrome o ubiquinol oxidase subunit IV
	VpaChn25_RS07260	0.493	Manganese-dependent inorganic pyrophosphatase
	VpaChn25_RS21575	2.081	Cytochrome c oxidase subunit II
	VpaChn25_RS21560	2.268	Cytochrome c oxidase subunit 3
	VpaChn25_RS21565	2.276	Cytochrome c oxidase assembly protein
	VpaChn25_RS02145	2.280	Ubiquinol-cytochrome c reductase iron-sulfur subunit
	VpaChn25_RS04445	2.456	Succinate dehydrogenase cytochrome b556 subunit
	VpaChn25_RS04450	2.464	Succinate dehydrogenase, hydrophobic membrane anchor protein
	VpaChn25_RS02155	2.911	Cytochrome c1
	VpaChn25_RS21540	3.411	COX15/CtaA family protein
	VpaChn25_RS02150	4.053	Cytochrome bc complex cytochrome subunit
Histidine metabolism	*VpaChn25_RS04675*	0.436	Aromatic amino acid ammonia-lyase
	VpaChn25_RS06765	3.843	Histidine ammonia-lyase
	VpaChn25_RS06780	4.142	Imidazolonepropionase
	VpaChn25_RS06770	4.374	Urocanate hydratase
	VpaChn25_RS06775	4.716	Formimidoylglutamase
Nitrogen metabolism	*VpaChn25_RS02355*	0.096	Glutamate synthase subunit β
	VpaChn25_RS02360	0.112	Glutamate synthase large subunit
	VpaChn25_RS20680	0.231	ABC transporter ATP-binding protein
	VpaChn25_RS16925	0.362	Carbonic anhydrase family protein
	VpaChn25_RS02345	0.473	FAD-dependent oxidoreductase
	VpaChn25_RS22655	0.481	Carbonic anhydrase
	VpaChn25_RS09500	3.477	Ammonia-forming nitrite reductase cytochrome c552 subunit
Amino sugar and nucleotide sugar	*VpaChn25_RS21110*	0.001	UDP-glucose 4-epimerase GalE

metabolism			
	VpaChn25_RS21105	0.001	UDP-glucose-hexose-1-phosphate uridylyltransferase
	VpaChn25_RS21100	0.001	Galactokinase
	VpaChn25_RS23815	0.164	N-acetylmuramic acid 6-phosphate etherase
	VpaChn25_RS07740	2.008	N-acetylglucosamine kinase
	VpaChn25_RS10005	2.279	NAD-dependent epimerase
	VpaChn25_RS12610	3.364	N, N'-diacetylchitobiose phosphorylase
	VpaChn25_RS12600	3.554	N-acetylglucosamine kinase
	VpaChn25_RS12605	5.481	Family 20 glycosylhydrolase
Nitrotoluene degradation	*VpaChn25_RS21015*	0.009	Alkene reductase
	VpaChn25_RS21020	0.021	Alkene reductase
	VpaChn25_RS23085	0.274	Oxygen-insensitive NAD(P)H nitroreductase
Taurine and hypotaurine metabolism	*VpaChn25_RS15390*	2.136	γ-glutamyltransferase
Propanoate metabolism	*VpaChn25_RS21225*	0.216	Acetate/propionate family kinase
	VpaChn25_RS10295	0.418	Phosphate acetyltransferase
	VpaChn25_RS20810	2.677	Pyruvate dehydrogenase (acetyl-transferring) E1 component subunit α
	VpaChn25_RS20820	2.715	2-oxo acid dehydrogenase subunit E2
	VpaChn25_RS20815	3.263	α-ketoacid dehydrogenase subunit β
Pyruvate metabolism	*VpaChn25_RS05195*	0.162	Formate C-acetyltransferase
	VpaChn25_RS14110	0.342	Phosphoenolpyruvate carboxylase
	VpaChn25_RS19425	0.377	Acetyl-CoA carboxylase, carboxyltransferase subunit β
	VpaChn25_RS12745	0.379	Pyruvate dehydrogenase complex dihydrolipoyllysine-residue acetyltransferase
	VpaChn25_RS18565	0.421	Thiolase family protein
Longevity regulating pathway	*VpaChn25_RS14590*	0.155	Chaperonin GroEL
	VpaChn25_RS03265	0.269	Molecular chaperone DnaK
	VpaChn25_RS17350	0.433	Catalase
	VpaChn25_RS17235	0.455	Chaperonin GroEL

Table S4. Major changed metabolic pathways in the Δ*VpaChn25_0714* mutant.

**Metabolism Pathway**	**Gene ID**	**Fold Change**	**Gene Description**
Propanoate metabolism	*VpaChn25_RS21225*	0.233	Acetate/propionate family kinase
	VpaChn25_RS07255	0.312	4-aminobutyrate-2-oxoglutarate transaminase
	VpaChn25_RS18570	0.373	CoA-acylating methylmalonate-semialdehyde dehydrogenase
	VpaChn25_RS10295	0.433	Phosphate acetyltransferase
	VpaChn25_RS19605	0.435	Iron-containing alcohol dehydrogenase
	VpaChn25_RS04475	2.019	ADP-forming succinate-CoA ligase subunit β
Mannose and fructose metabolism	*VpaChn25_RS19525*	0.080	1-phosphofructokinase
	VpaChn25_RS21725	0.426	Zinc-binding dehydrogenase
	VpaChn25_RS20215	3.217	Mannose-6-phosphate isomerase, class I
Nitrotoluene degradation	*VpaChn25_RS17690*	0.325	Alkene reductase
	VpaChn25_RS18635	0.374	NAD(P)H-dependent oxidoreductase
	VpaChn25_RS23085	0.416	Oxygen-insensitive NAD(P)H nitroreductase
	VpaChn25_RS19315	0.439	Alkene reductase
Phosphotransferase system (PTS)	*VpaChn25_RS19520*	0.047	PTS fructose transporter subunit IIBC
	VpaChn25_RS19530	0.134	Fused PTS fructose transporter subunit IIA / HPr protein
	VpaChn25_RS17315	0.187	PTS sugar transporter subunit IIA
	VpaChn25_RS21730	0.265	PTS mannitol transporter subunit IICB
	VpaChn25_RS16970	0.343	PTS sugar transporter subunit IIB
	VpaChn25_RS16965	0.351	PTS ascorbate transporter subunit IIC
	VpaChn25_RS21735	0.357	PTS sugar transporter subunit IIA

	VpaChn25_RS23820	0.496	PTS N-acetylmuramic acid transporter subunit IIBC
	VpaChn25_RS23450	2.823	Glucose-specific PTS transporter subunit IIBC
Lysine degradation	*VpaChn25_RS14795*	0.052	Lysine decarboxylase CadA
	VpaChn25_RS07250	0.205	NAD-dependent succinate-semialdehyde Dehydrogenase
Glycolysis/Gluconeogenesis	*VpaChn25_RS10590*	0.228	D-hexose-6-phosphate mutarotase
	VpaChn25_RS23575	0.237	6-phospho-β-glucosidase
	VpaChn25_RS10585	0.285	Type I glyceraldehyde-3-phosphate dehydrogenase
	VpaChn25_RS10475	0.396	Bifunctional acetaldehyde-CoA/alcohol dehydrogenase
	VpaChn25_RS19575	0.406	Pyruvate kinase
	VpaChn25_RS01325	0.483	Triose-phosphate isomerase
	VpaChn25_RS00605	2.022	Phosphoenolpyruvate carboxykinase (ATP)
	VpaChn25_RS08880	2.211	Aldehyde dehydrogenase
	VpaChn25_RS18050	2.964	Glyceraldehyde-3-phosphate dehydrogenase
Benzoate degradation	*VpaChn25_RS18485*	0.463	Carboxymuconolactone decarboxylase family protein
Pyruvate metabolism	*VpaChn25_RS19425*	0.166	Acetyl-CoA carboxylase, carboxyltransferase subunit β
	VpaChn25_RS05195	0.172	Formate C-acetyltransferase
	VpaChn25_RS19320	0.298	Lactoylglutathione lyase
	VpaChn25_RS01820	0.405	2-isopropylmalate synthase
	VpaChn25_RS14110	0.490	Phosphoenolpyruvate carboxylase
	VpaChn25_RS18565	0.494	Thiolase family protein
	VpaChn25_RS08505	2.146	Acylphosphatase
	VpaChn25_RS14715	2.204	Acetate-CoA ligase
Ascorbate and aldarate metabolism	*VpaChn25_RS07015*	0.151	Aldehyde dehydrogenase ($NADP^+$)
	VpaChn25_RS10005	2.710	NAD-dependent epimerase
Butanoate metabolism	*VpaChn25_RS17675*	0.268	SDR family oxidoreductase
	VpaChn25_RS15645	0.445	Acetolactate synthase 2 catalytic subunit
	VpaChn25_RS18170	2.017	SDR family oxidoreductase
Fatty acid degradation	*VpaChn25_RS18430*	0.470	Long-chain fatty acid-CoA ligase
	VpaChn25_RS10820	2.100	Fatty acid oxidation complex subunit α FadJ
	VpaChn25_RS10825	2.324	Acetyl-CoA C-acyltransferase FadI
	VpaChn25_RS18175	2.425	Acetyl-CoA C-acetyltransferase
Histidine metabolism	*VpaChn25_RS04675*	0.307	Aromatic amino acid ammonia-lyase
	VpaChn25_RS06765	3.400	Histidine ammonia-lyase
	VpaChn25_RS06780	3.435	Imidazolonepropionase
	VpaChn25_RS06770	4.504	Urocanate hydratase
	VpaChn25_RS06775	4.629	Formimidoylglutamase
β-lactam resistance	*VpaChn25_RS07075*	0.119	ATP-binding cassette domain-containing protein
	VpaChn25_RS07085	0.214	ABC transporter permease subunit
	VpaChn25_RS07090	0.306	Oligopeptide ABC transporter permease OppB
	VpaChn25_RS12510	0.357	Porin
	VpaChn25_RS07080	0.417	ATP-binding cassette domain-containing protein
	VpaChn25_RS07095	0.426	Peptide ABC transporter substrate-binding protein
	VpaChn25_RS21835	0.432	Carbenicillin-hydrolyzing class A β-lactamase CARB-18
	VpaChn25_RS18245	0.462	Multidrug efflux RND transporter periplasmic adaptor subunit VmeY

Table S5. Major changed metabolic pathways in the Δ*VpaChn25_RS25055* mutant.

**Metabolism Pathway**	**Gene ID**	**Fold Change**	**Gene Description**
Sulfur metabolism	*VpaChn25_RS07520*	0.298	Dimethylsulfoxide reductase subunit B
	VpaChn25_RS07525	0.319	Dimethyl sulfoxide reductase anchor subunit
	VpaChn25_RS07515	0.343	Dimethyl sulfoxide reductase subunit A
	VpaChn25_RS04975	0.396	PLP-dependent cysteine synthase family protein
	VpaChn25_RS06515	0.487	C-type cytochrome
	VpaChn25_RS09955	2.050	Polysulfide reductase NrfD
	VpaChn25_RS01585	2.714	Sulfate adenylyltransferase subunit CysN

	VpaChn25_RS01580	3.078	Sulfate adenylyltransferase subunit CysD
Glyoxylate and dicarboxylate metabolism	*VpaChn25_RS17350*	0.436	Catalase
	VpaChn25_RS21805	2.591	Formate dehydrogenase subunit α
	VpaChn25_RS07825	3.661	Formate dehydrogenase FDH3 subunit β
	VpaChn25_RS07820	3.982	Formate dehydrogenase subunit α
	VpaChn25_RS07830	4.105	Formate dehydrogenase subunit γ
	VpaChn25_RS00565	6.026	Glutamate-ammonia ligase
Arginine biosynthesis	*VpaChn25_RS11620*	0.084	Amino-acid N-acetyltransferase
	VpaChn25_RS14100	0.100	N-acetyl-γ-glutamyl-phosphate reductase
	VpaChn25_RS14095	0.107	Acetylglutamate kinase
	VpaChn25_RS13555	0.302	Ornithine carbamoyltransferase
	VpaChn25_RS14090	0.305	Argininosuccinate synthase
Longevity regulating pathway	*VpaChn25_RS14590*	0.290	Chaperonin GroEL
	VpaChn25_RS17235	0.313	Chaperonin GroEL
	VpaChn25_RS03265	0.373	Molecular chaperone DnaK
	VpaChn25_RS20830	0.472	Glutathione S-transferase family protein
Glycolysis/Gluconeogenesis	*VpaChn25_RS10590*	0.215	D-hexose-6-phosphate mutarotase
	VpaChn25_RS10585	0.363	Type I glyceraldehyde-3-phosphate dehydrogenase
	VpaChn25_RS08880	0.368	Aldehyde dehydrogenase
	VpaChn25_RS21435	0.420	L-threonine dehydrogenase
	VpaChn25_RS23575	0.426	6-phospho-β-glucosidase
	VpaChn25_RS10475	0.449	Bifunctional acetaldehyde-CoA/alcohol dehydrogenase
	VpaChn25_RS18050	2.379	Glyceraldehyde-3-phosphate dehydrogenase
	VpaChn25_RS01350	2.553	Class II fructose-bisphosphatase
NOD-like receptor signaling pathway	*VpaChn25_RS04175*	0.317	Flagellin
	VpaChn25_RS04340	0.389	Molecular chaperone HtpG
	VpaChn25_RS11070	0.396	Flagellin
	VpaChn25_RS11075	0.451	Flagellin
Ribosome	*VpaChn25_RS01405*	2.006	50S ribosomal protein L3
	VpaChn25_RS01475	2.016	30S ribosomal protein S8
	VpaChn25_RS14000	2.028	30S ribosomal protein S18
	VpaChn25_RS01415	2.052	50S ribosomal protein L23
	VpaChn25_RS01520	2.054	30S ribosomal protein S11
	VpaChn25_RS01465	2.094	50S ribosomal protein L5
	VpaChn25_RS01735	2.100	50S ribosomal protein L21
	VpaChn25_RS02135	2.191	50S ribosomal protein L13
	VpaChn25_RS01450	2.206	30S ribosomal protein S17
	VpaChn25_RS14165	2.253	30S ribosomal protein S7
	VpaChn25_RS01460	2.357	50S ribosomal protein L24
	VpaChn25_RS02140	2.478	30S ribosomal protein S9
	VpaChn25_RS01740	2.705	50S ribosomal protein L27
Monobactam biosynthesis	*VpaChn25_RS02405*	0.288	Bifunctional a spartate kinase/homoserine dehydrogenase I
	VpaChn25_RS13865	2.289	Lysine-sensitive a spartokinase 3

Table S6. Major changed metabolic pathways in the Δ*VpaChn25_RS25055-0713-0714* mutant.

**Metabolism Pathway**	**Gene ID**	**Fold Change**	**Gene Description**
Mannose and fructose metabolism	*VpaChn25_RS19525*	0.095	1-phosphofructokinase
	VpaChn25_RS22285	0.456	Mannose-6-phosphate isomerase, class I
	VpaChn25_RS01930	0.499	Mannitol-1-phosphate 5-dehydrogenase
Propanoate metabolism	*VpaChn25_RS05195*	0.055	Formate C-acetyltransferase
	VpaChn25_RS21225	0.170	Acetate/propionate family kinase
	VpaChn25_RS10295	0.234	Phosphate acetyltransferase
	VpaChn25_RS10290	0.457	Acetate kinase

	VpaChn25_RS04475	2.407	ADP-forming succinate-CoA ligase subunit β
	VpaChn25_RS20820	2.637	2-oxo acid dehydrogenase subunit E2
	VpaChn25_RS20810	2.744	Pyruvate dehydrogenase (acetyl-transferring) E1 component subunit α
	VpaChn25_RS20815	3.264	α-ketoacid dehydrogenase subunit β
Alanine, aspartate and glutamate metabolism	*VpaChn25_RS02355*	0.098	Glutamate synthase subunit β
	VpaChn25_RS02360	0.100	Glutamate synthase large subunit
	VpaChn25_RS16990	0.160	Aspartate aminotransferase family protein
	VpaChn25_RS01880	0.237	Glutamine-fructose-6-phosphate transaminase (isomerizing)
	VpaChn25_RS13400	0.476	NAD-dependent succinate-semialdehyde dehydrogenase
	VpaChn25_RS04365	0.480	Asparagine synthase B
	VpaChn25_RS13565	2.013	Aspartate carbamoyltransferase regulatory subunit
	VpaChn25_RS02295	2.104	Carbamoyl-phosphate synthase large subunit
	VpaChn25_RS02290	2.208	Glutamine-hydrolyzing carbamoyl-phosphate synthase small subunit
	VpaChn25_RS17645	2.878	Adenylosuccinate synthase
NOD-like receptor signaling pathway	*VpaChn25_RS11075*	0.331	Flagellin
	VpaChn25_RS11070	0.340	Flagellin
	VpaChn25_RS04170	0.417	Flagellin
	VpaChn25_RS04340	0.419	Molecular chaperone HtpG
	VpaChn25_RS11085	0.419	Flagellin
	VpaChn25_RS04175	0.425	Flagellin
PTS	*VpaChn25_RS19520*	0.056	PTS fructose transporter subunit IIBC
	VpaChn25_RS19530	0.122	Fused PTS fructose transporter subunit IIA/HPr protein
	VpaChn25_RS22280	0.162	Fructose-specific PTS transporter subunit EIIC
	VpaChn25_RS17315	0.192	PTS sugar transporter subunit IIA
	VpaChn25_RS21735	0.346	PTS sugar transporter subunit IIA
	VpaChn25_RS21730	0.374	PTS mannitol transporter subunit IICB
	VpaChn25_RS01935	0.392	PTS mannitol transporter subunit IICBA
	VpaChn25_RS04195	0.405	HPr family phosphocarrier protein
	VpaChn25_RS23450	2.104	Glucose-specific PTS transporter subunit IIBC
	VpaChn25_RS13475	2.207	PTS sugar transporter subunit IIB
	VpaChn25_RS13470	2.814	PTS sugar transporter subunit IIC
Amino sugar and nucleotide sugar metabolism	*VpaChn25_RS11760*	0.427	UDP-glucose 4-epimerase GalE
	VpaChn25_RS11755	0.435	UDP-glucose-hexose-1-phosphate uridylyltransferase
	VpaChn25_RS12480	0.441	Phosphoglucosamine mutase
	VpaChn25_RS21110	0.452	UDP-glucose 4-epimerase GalE
	VpaChn25_RS21100	0.472	Galactokinase
	VpaChn25_RS21105	0.498	UDP-glucose-hexose-1-phosphate uridylyltransferase
	VpaChn25_RS11455	2.100	Ig-like domain-containing protein
	VpaChn25_RS10005	2.435	NAD-dependent epimerase
	VpaChn25_RS12610	3.486	N, N'-diacetylchitobiose phosphorylase
	VpaChn25_RS12600	5.279	N-acetylglucosamine kinase
	VpaChn25_RS12605	5.692	Family 20 glycosylhydrolase
Arginine and proline metabolism	*VpaChn25_RS07035*	0.189	Ornithine cyclodeaminase family protein
	VpaChn25_RS23295	0.310	Ornithine decarboxylase SpeF
	VpaChn25_RS07215	0.393	Glutamine synthetase family protein
	VpaChn25_RS16685	2.105	Arginine decarboxylase
	VpaChn25_RS09600	2.229	Carboxynorspermidine decarboxylase
	VpaChn25_RS14270	2.285	Succinylglutamate-semialdehyde dehydrogenase
	VpaChn25_RS14275	2.301	Arginine N-succinyltransferase
	VpaChn25_RS09605	2.957	Saccharopine dehydrogenase family protein
	VpaChn25_RS23720	3.110	Bifunctional proline dehydrogenase/L-glutamate γ-semialdehyde dehydrogenase PutA
Oxidative phosphorylation	*VpaChn25_RS02155*	2.218	Cytochrome c1

	VpaChn25_RS20880	2.544	Cytochrome o ubiquinol oxidase subunit IV
	VpaChn25_RS07945	2.564	CcoQ/FixQ family Cbb3-type cytochrome c oxidase assembly chaperone
	VpaChn25_RS20890	2.569	Cytochrome o ubiquinol oxidase subunit I
	VpaChn25_RS21570	2.638	Cytochrome c oxidase subunit I
	VpaChn25_RS21575	2.724	Cytochrome c oxidase subunit II
	VpaChn25_RS02150	2.740	Cytochrome bc complex cytochrome b subunit
	VpaChn25_RS21535	2.849	Heme o synthase
	VpaChn25_RS21560	2.981	Cytochrome c oxidase subunit 3
	VpaChn25_RS21540	3.028	COX15/CtaA family protein
	VpaChn25_RS20875	3.328	Heme o synthase
	VpaChn25_RS20885	4.543	Cytochrome o ubiquinol oxidase subunit III
	VpaChn25_RS21565	5.684	Cytochrome c oxidase assembly protein
Glycolysis/Gluconeogenesis	*VpaChn25_RS10590*	0.166	D-hexose-6-phosphate mutarotase
	VpaChn25_RS10585	0.186	Type I glyceraldehyde-3-phosphate dehydrogenase
	VpaChn25_RS23575	0.228	6-phospho-β-glucosidase
	VpaChn25_RS10475	0.286	Bifunctional acetaldehyde-CoA/alcohol dehydrogenase
	VpaChn25_RS14485	0.382	2, 3-bisphosphoglycerate-independent phosphoglycerate mutase
	VpaChn25_RS01325	0.457	Triose-phosphate isomerase
	VpaChn25_RS19575	0.467	Pyruvate kinase
	VpaChn25_RS18050	4.409	Glyceraldehyde-3-phosphate dehydrogenase
	VpaChn25_RS08880	7.179	Aldehyde dehydrogenase
Thiamine metabolism	*VpaChn25_RS15440*	2.127	2-iminoacetate synthase ThiH
	VpaChn25_RS16405	2.192	Thiamine phosphate synthase
	VpaChn25_RS15445	2.476	Thiazole synthase
	VpaChn25_RS16410	2.520	Hydroxyethylthiazole kinase
	VpaChn25_RS15455	2.602	Thiazole biosynthesis adenylyltransferase ThiF
	VpaChn25_RS15460	2.623	Thiamine phosphate synthase
	VpaChn25_RS16415	3.115	Thiaminase II
Arginine biosynthesis	*VpaChn25_RS11620*	0.101	Amino-acid N-acetyltransferase
	VpaChn25_RS14100	0.158	N-acetyl-γ-glutamyl-phosphate reductase
	VpaChn25_RS14095	0.204	Acetylglutamate kinase
	VpaChn25_RS14090	0.329	Argininosuccinate synthase
	VpaChn25_RS13555	0.340	Ornithine carbamoyltransferase
	VpaChn25_RS14085	0.376	Argininosuccinate lyase
